# GPR15 differentially regulates the effects of cigarette smoke exposure on Crohn’s disease and ulcerative colitis

**DOI:** 10.1038/s41392-025-02384-8

**Published:** 2025-09-17

**Authors:** Luhua Gao, Huaping Zheng, Qing Zhao, Yubin Wang, Yong He, Can Hou, Na Yang, Kun Liang, Wenjian Meng, Xuefei He, Kun Zheng, Wenning Tian, Jiacheng Zhang, Ting Zhang, Hui Mao, Liming Zhang, Jingyu Zhang, Jingqiu Cheng, Jürgen Brosius, Huan Song, Yuchuan Huang, Yi Chen, Cheng Deng

**Affiliations:** 1 Department of Respiratory and Critical Care Medicine, Center for High Altitude Medicine, National Clinical Research Center for Geriatrics, West China Hospital, Sichuan University, Chengdu, China; 2 Department of Laboratory Medicine, West China Hospital, Sichuan University, Chengdu, China; 3 West China Biomedical Big Data Center, West China Hospital, Sichuan University, Chengdu, China; 4 Med-X Center for Informatics, Sichuan University, Chengdu, China; 5 Department of Biological Products, Institute of Drug Development, Chengdu Di’ao Pharmaceutical Group Co., Ltd, Chengdu, China; 6 Harmful Components and Tar Reduction in Tobacco Key Laboratory of Sichuan Province, Chengdu, China; 7 Division of Gastrointestinal Surgery and Colorectal Cancer Center, Department of General Surgery, West China Hospital, Sichuan University, Chengdu, China; 8 Division of Thoracic Surgery, NO.3 Affiliated Hospital of Chengdu University of Traditional Chinese Medicine (West District), Chengdu, China; 9 Department of Gastroenterology, Tangdu Hospital, Fourth Military Medical University, Xi’an, China; 10 Center for High Altitude Medicine, Qinghai, 810000 China

**Keywords:** Gastrointestinal diseases, Immunological disorders

## Abstract

Inflammatory bowel disease (IBD), which includes Crohn’s disease (CD) and ulcerative colitis (UC), is a chronic disorder characterized by gastrointestinal inflammation. Cigarette smoke is a well-established risk factor for the development and exacerbation of CD while exerting a paradoxical protective effect against the onset of UC. The exact mechanisms by which cigarette smoke influences IBD, as well as the opposite effects in UC and CD, have long remained unexplained. Here, we demonstrated the detrimental impact of cigarette smoke on CD progression while highlighting its beneficial effects on UC, as evidenced by analyses of human sample data. Mouse models of CD and UC exposed to cigarette smoke presented phenotypes consistent with those observed in human disease. GPR15, previously reported to direct regulatory T (Treg) cell colon homing, was upregulated in the colon tissues of both chemically induced colitis models after smoke exposure. Importantly, *Gpr15* deletion ameliorated smoke-induced CD while increasing UC severity in mice. Furthermore, our study revealed that cigarette smoke mediated GPR15 to amplify colonic T helper type 17 (Th17) cell populations, thereby worsening the adverse effects of smoking on CD in mouse models. Moreover, cigarette smoke induced an increase in Treg cells through GPR15, which contributed to mitigating its impact on UC in mouse models. Moreover, in cigarette smoke-exposed CD and UC model mice, C57BL/6JGpt-Tg (human *GPR15*) transgenic mice presented phenotypes opposite those of *Gpr15-*deficient mice. Overall, our study offers mechanistic insights into the role of cigarette smoke-induced GPR15^+^ T cells in mediating the divergent effects of smoking on UC and CD.

## Introduction

Inflammatory bowel disease (IBD) refers to disorders characterized by persistent inflammation of the gastrointestinal tract, predominantly manifesting as either Crohn’s disease (CD) or ulcerative colitis (UC).^1^ Despite their shared characteristics, each disorder has distinct clinical manifestations and pathological profiles.^2^ The etiology of IBD has not been fully elucidated; however, its pathogenesis is thought to stem from a genetic predisposition resulting in dysregulated interactions between the immune system and environmental factors.^3^ Environmental risk factors, including cigarette smoking, substance abuse, psychological stress, and poor dietary habits, are recognized as contributors to IBD pathogenesis.^4^ Cigarette smoke is well known as a potent inducer of chronic pulmonary inflammation, as evidenced by significant immune cell infiltration, and is also a major environmental risk factor for IBD.^5^ Moreover, retrospective observational studies have underscored the conflicting effects of tobacco consumption on both the progression and clinical presentation of IBD.^6^ In individuals with CD, cigarette smoke has been consistently linked to a more aggressive disease course, unfavorable clinical outcomes, and a heightened risk of postoperative recurrence.^7^ Additionally, smoking has been implicated in the progression of the disease from an inflammatory phenotype to a penetrating or fibrotic stenosis phenotype.^8^ In contrast to CD, cigarette smoke does not negatively impact clinical outcomes in patients with established UC and may be protective against disease risk, progression, relapse, and adverse outcomes.^9^ Accordingly, smoking cessation increases the risk of a UC flare, whereas CD patients are more likely to experience a decrease in disease severity.^10^ The underlying mechanisms likely involve diverse constituents of smoke, including nicotine, oxygen free radicals, and carbon monoxide, which may affect various targets, such as the mucus layer, gut microbiota, immune cells, gastrointestinal motility, and microvasculature.^11^ However, the precise mechanisms by which cigarette smoke influences IBD, along with the differential effects observed in UC and CD, remain poorly understood.

Disrupted immune cell trafficking and the formation of pathogenic immune cell networks are central to mucosal inflammation and tissue damage in IBD.^12^ T-cell subtypes are pivotal in detecting inflammation and regulating precise, localized immune responses, which are frequently impaired in IBD.^13^ Th17 cells are a subset of CD4^+^ T cells that differentiate in an RORγt-dependent manner to produce interleukin (IL)-17A and IL-17F, contributing critically to host defense and the pathogenesis of inflammatory diseases.^14^ Recent research has demonstrated that Th17 cells are pivotal in the pathogenesis of IBD, contributing to both mucosal barrier disruption and the amplification of inflammatory responses.^15^ Other T-cell subsets, such as Treg cells characterized by FOXP3 expression, are essential for maintaining immune homeostasis.^16^ In IBD, they play a critical role by secreting anti-inflammatory cytokines, including IL-10 and transforming growth factor-beta (TGF-β), thus suppressing effector immune cell activity and regulating inflammatory responses.^17^

Among the molecules involved in regulating T-cell homing and function, G protein-coupled receptor 15 (GPR15) has recently received considerable attention.^18^ GPR15, which is highly expressed on certain T-cell subsets, functions not only as a coreceptor for human immunodeficiency virus (HIV) and simian immunodeficiency virus (SIV) but also as a critical mucosal homing receptor that mediates the migration of T cells to inflamed intestinal tissues.^19^ Recent studies have demonstrated that cigarette smoking induces hypomethylation of the *GPR15* gene locus, leading to significant upregulation of *GPR15* mRNA expression.^20^ Specifically, current smokers present expression levels that are approximately 7.6 times higher than those observed in never smokers.^21^ Furthermore, in individuals with multiple sclerosis who smoke, there is an increased frequency of GPR15^+^ CD4^+^ T cells exhibiting Th17-like characteristics, implicating GPR15 as a potential link between environmental exposure and autoimmune pathogenesis.^22^

Building on extensive evidence, particularly the established relationship between cigarette smoking, GPR15 upregulation, and T-cell function and trafficking, we hypothesized that GPR15^+^ T cells play a pivotal role in mediating the differential impact of cigarette smoke on the pathogenesis of CD and UC. Therefore, we focused on characterizing the involvement of GPR15^+^ T cells in murine models of IBD induced by cigarette smoke exposure. In this study, we observed that in wild-type mouse models of CD induced by 2,4,6-trinitrobenzene sulfonic acid (TNBS), exposure to cigarette smoke significantly exacerbated intestinal inflammation. Conversely, in dextran sulfate sodium (DSS)-induced UC models, cigarette smoke exposure notably alleviated symptoms of colitis. Additionally, in *Gpr15*-deficient mice, cigarette smoke exposure reduced the severity of TNBS-induced colitis, whereas in DSS-induced *Gpr15*-deficient UC models, it re-resulted in more severe disease progression. Compared with wild-type (WT) mice subjected to identical treatment conditions, C57BL/6JGpt-Tg (human *GRP15*) transgenic mice presented a contrasting phenotype of colitis following exposure to cigarette smoke. In TNBS-induced CD-like colitis, cigarette smoke upregulates *GPR15* on T cells, promoting Th17 differentiation and aggravating the disease. DSS-induced UC-like colitis similarly increases *GPR15* but drives Treg differentiation, alleviating disease severity. Our study offers mechanistic insights into the crucial role of cigarette smoke-induced GPR15^+^ T cells in mediating the divergent effects of smoking on CD and UC.

## Results

### Different impacts of cigarette smoke on IBD outcomes

The impact of smoking on patients with CD and UC has yielded inconsistent results, partly due to the lack of reliable studies involving sufficiently large cohorts. To elucidate the associations between smoking status—including never, ever, previous, and current smokers—and the risk of CD and UC, data from the UK Biobank were systematically analyzed. To uncover the relationship between smoking habits and the progression of IBD, we applied an adjusted Cox regression model to estimate the hazard ratios (HRs) and their corresponding 95% confidence intervals (CIs), contrasting never smokers with previous and current smokers among patients diagnosed with CD and UC. Among the 2,120 patients with CD whose data were complete, the HRs for ever smoking, previous smoking, and current smoking were 1.2719 (95% CI: 1.1636–1.3903, *p* = 1.19e-7), 1.2655 (95% CI: 1.1554–1.3869, *p* = 3.98e-7) and 1.3707 (95% CI: 1.2114–1.5509, *p* = 5.62e-07), respectively, greater than those of never smokers. With respect to the 4,161 UC samples analyzed, the HRs of ever smoking and previous smoking were 1.2084 (95% CI: 1.1324–1.2894, *p* = 1.11e-08) and 1.4795 (95% CI: 1.3897–1.5762, *p* = 2.00e-16), respectively. Interestingly, within the UC group, current smoking presented a paradoxical protective effect, with an HR of 0.7569 (95% CI: 0.6788–0.844, *p* = 5.36e-7) compared to never-smoking individuals (Supplementary Table 1). To further explore genetic contributions, SNPs near GPR15 and their interactions with smoking were included. Although several SNPs showed significant effects, adjustment for these variants had only a minor impact on risk estimates and did not alter the main associations (Supplementary Table 2). Therefore, smoking serves as a significant risk factor for CD while providing a paradoxical protective effect in UC.

**Fig. 1:**
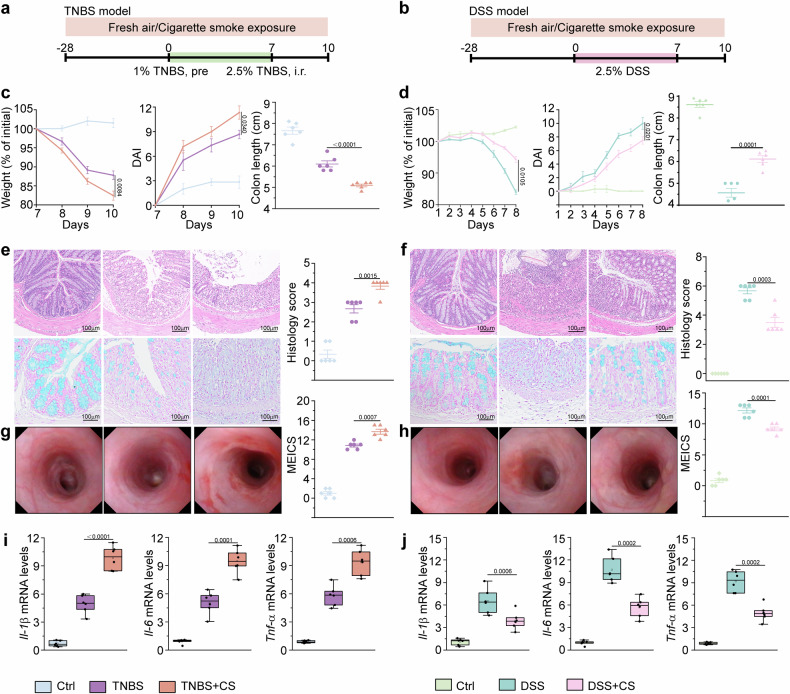
Divergent impact of cigarette smoke on disease phenotypes in IBD models. **a** Diagram of a colitis mouse model induced with TNBS, with exposure to cigarette smoke or fresh air as a control. **b** Schematic of a colitis mouse model induced by DSS, with exposure to cigarette smoke or fresh air as a control. **c** Percent changes in body weight, disease activity index (DAI) scores, and colon length in the TNBS model group. **d** Percent changes in body weight, DAI scores, and colon length in the DSS model group. **e** Representative images of colon sections from the TNBS model: H&E staining (top left), Alcian blue staining (left middle), and histology score (right of the left panel). **f** Representative images of colon sections from the DSS model: H&E staining (top right), Alcian blue staining (right middle), and histology score (rightmost panel). **g** Colonoscopy images of the TNBS group (bottom left) with corresponding MEICS scores (to the right of the left panel). **h** Colonoscopy images of the DSS group (right) with corresponding MEICS scores (rightmost panel). **i** mRNA expression levels of *Il-1β, Il-6*, and *Tnf-α* in the TNBS model group (left). **j** mRNA expression levels of *Il-1β*, *Il-6*, and *Tnf-α* in the DSS model group (right). Scale bar, 100 μm. *n* = six male mice per group. The data are presented as the means ± SEMs; *p < 0.05, **p < 0.01, and *p < 0.001 according to two-way ANOVA with multiple comparisons. All the experiments were repeated three times and yielded consistent results

We evaluated the impact of cigarette smoke on the severity of acute colitis induced by TNBS and DSS in wild-type C57BL/6 mice (Fig. 1a, b). Compared with control mice, TNBS-treated mice presented severe colitis symptoms, such as continuous weight loss, increased disease activity index (DAI) scores, and reduced colon length, with significant tissue damage confirmed by histopathological and colonoscopic analyses (Fig. 1c, e, g, and Supplementary Fig. 1a). Cotreatment with TNBS and cigarette smoke exacerbated these effects, leading to increased weight loss, elevated DAI scores, shortened colon length, additional tissue and mucosal damage evident from histopathological analysis, and increased murine endoscopic index of colitis severity (MEICS) (Fig. 1c, e, g and Supplementary Fig. 1a). Cigarette smoke exposure combined with TNBS treatment significantly increased *Il-1β*, *Il-6*, and *Tnf-α* mRNA levels in colon tissue, indicating increased inflammation (Fig. 1i).

Compared with control mice, DSS-treated mice presented increased weight loss, greater disease activity, greater colon shortening, and aggravated histopathological damage (Fig. 1d, f and Supplementary Fig. 1c). Compared with those treated with DSS alone, mice coexposed to cigarette smoke and DSS demonstrated significantly greater relief from colitis symptoms. This coexposure led to improved weight recovery, reduced DAI scores, prevention of colon shortening, and decreased tissue damage scores (Fig. 1d, f and Supplementary Fig. 1c). The colonoscopy results confirmed significant improvements in DSS-induced inflammation in the mice exposed to cigarette smoke (Fig. 1h). Cigarette smoke exposure combined with DSS treatment resulted in significantly lower levels of *Il-1β*, *Il-6*, and *Tnf-α* mRNA in colon tissue, indicating a reduction in the inflammatory response (Fig. 1j). Additionally, four weeks of cigarette smoke exposure significantly increased urinary cotinine levels, indicating substantial systemic absorption (Supplementary Fig. 1b, d). These results are consistent with those of previous studies, indicating that cigarette smoke exposure may worsen inflammation in TNBS-induced colitis, a CD model, but alleviate colonic inflammation and aid recovery in DSS-induced colitis, a UC model.

### The conflicting impact of cigarette smoke on IBD subsets is attributed to the expression of *Gpr15*

**Fig. 2:**
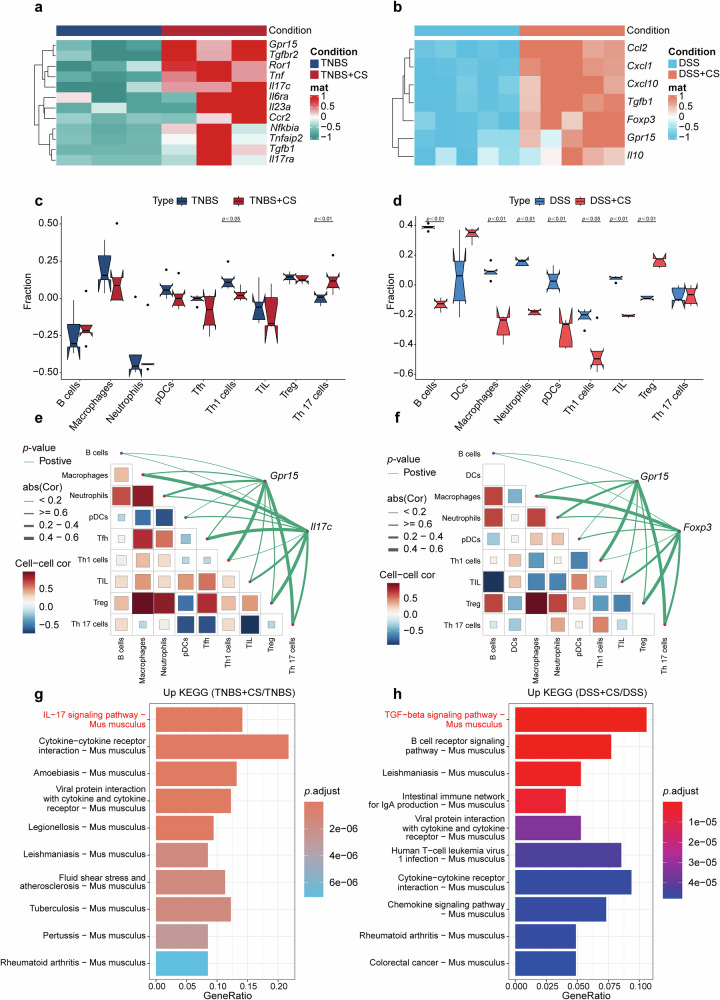
Differential gene expression and immune cell infiltration analysis in IBD subtypes. **a** Heatmaps illustrating the differential gene expression between TNBS with cigarette smoke exposure and TNBS alone (*n* = 3). **b** Heatmaps showing the differential gene expression between the DSS with cigarette smoke exposure group and the DSS control group (*n* = 5). Each row represents a gene, and each column corresponds to a sample. Red highlights denote increased expression levels, whereas blue denotes decreased expression levels relative to the average. **c** Comparison of the fraction of immune cell types between TNBS exposed to cigarette smoke and TNBS alone. **d** Immune infiltration profiles comparing DSS with cigarette smoke exposure and DSS alone across various immune cell types. The x-axis lists the cell types, whereas the y-axis depicts the fraction of each cell type. The blue dots represent control samples, and the red dots denote the two colitis subtypes. **e** Correlation analysis between *Gpr15* and *Il17c* expression and immune cell infiltration under TNBS conditions, both with and without cigarette smoke exposure. **f** Correlation matrix of *Gpr15*, *Foxp3*, and immune cell infiltration under DSS conditions with and without cigarette smoke. **g** KEGG pathway analysis of upregulated shared gene sets comparing TNBS with cigarette smoke exposure to TNBS alone. **h** KEGG pathway analysis of upregulated shared gene sets comparing DSS with cigarette smoke exposure to DSS controls

To evaluate the impact of cigarette smoke on colitis in mice, we conducted RNA sequencing on TNBS- and DSS-induced colitis tissues postexposure. Heatmaps of differentially expressed genes revealed distinct responses between the TNBS and DSS models. In the TNBS group, smoke exposure increased the expression of proinflammatory genes such as *Il17c*, *Il17ra*, *Roc1*, *Tnf*, *Tgfbr2*, *Il6ra*, *Il23a*, and *Ccl12* (Fig. 2a). Conversely, the DSS group presented increased levels of immunosuppressive cytokines, such as *Foxp3*, *Il-10*, *Ccl2*, *Cxcl1*, and *Tgfb1* (Fig. 2b). Notably, both groups presented significant *Gpr15* upregulation after smoke exposure. Quantitative analysis revealed that cigarette smoke in conjunction with TNBS led to an increased ratio of Th17 cells without affecting Treg responses, in contrast with the results of the control groups treated with TNBS alone (Fig. 2c). Similarly, smoke exposure combined with DSS resulted in an elevated ratio of Treg cells without affecting Th17 populations, differing from the results of the control groups treated with DSS only (Fig. 2d).

Analysis of immune cell infiltration revealed that exposure to cigarette smoke combined with TNBS increased the associations between *Gpr15* and immune cells, particularly CD4^+^ effector T cells, which are focused on Th17 cells (Fig. 2e). Conversely, cigarette smoke exposure to DSS strongly linked *Gpr15* and Treg cells (Fig. 2f). KEGG analysis of the RNA-seq data revealed that the Th17 signaling pathway and cytokine‒cytokine receptor interactions were enriched in the TNBS-induced colitis postcigarette smoke group (Fig. 2g), whereas the anti-inflammatory role of the TGF‒beta signaling pathway was enriched in the DSS-induced colitis postcigarette smoke group (Fig. 2h). These results suggest that GPR15 plays a crucial role in modulating immune cell function across different colitis models following smoke exposure and that this regulation is closely linked to disease prognosis.

### Cigarette smoke exposure in *Gpr15*-deficient mice attenuates TNBS-induced colitis but exacerbates DSS-induced UC colitis

**Fig. 3:**
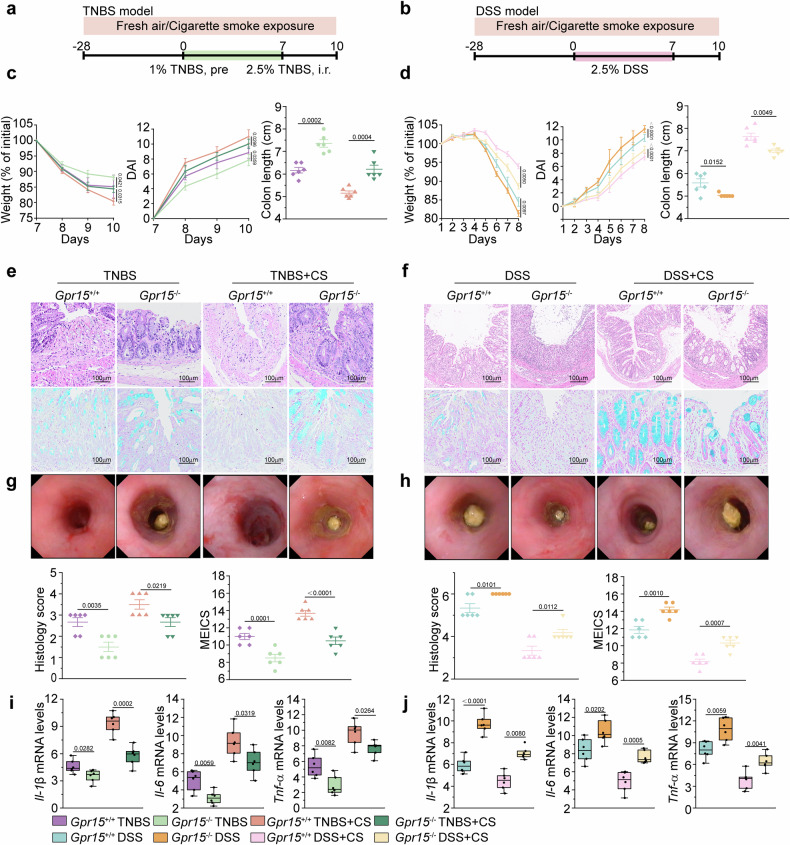
*Gpr15* deficiency in mice: cigarette smoke mitigates TNBS-induced colitis but exacerbates DSS-induced colitis. **a** Schematic overview of the *Gpr15*-deficient mouse model with cigarette smoke exposure and TNBS-induced colitis. **b** Schematic illustration of the *Gpr15*-deficient mouse model with cigarette smoke exposure and DSS-induced colitis. **c** Percent changes in body weight, DAI scores, and colon length in the TNBS-induced colitis model. **d** Percent changes in body weight, DAI scores, and colon length in the DSS-induced colitis model. **e** Representative colon section images for the TNBS model: top left—H&E staining, bottom left—Alcian blue staining, and bottom-left panel—Histology score. **f** Representative colon section images for the DSS model: top right—H&E staining, bottom right—Alcian blue staining, and bottom-right panel—Histology score. **g** Colonoscopy images from the TNBS group are shown on the left, and the corresponding MEICS scores are shown in the bottom-left panel. **h** Colonoscopy images from the DSS group are shown on the right, and the corresponding MEICS scores are shown in the bottom-right panel. **i** mRNA expression levels of *Il-1β*, *Il-6*, and *Tnf-α* in the TNBS model group are presented (left). **j** mRNA expression levels of *Il-1β*, *Il-6*, and *Tnf-α* in the DSS model group are presented (right). Scale bar, 100 μm. Each group consisted of six male mice. The data are presented as the means ± SEMs; *p < 0.05, **p < 0.01, and ***p < 0.001 according to two-way ANOVA with multiple comparisons. All the experiments were repeated three times independently and yielded consistent results

To investigate the role of *Gpr15* in UC and CD under cigarette smoke exposure, we used *Gpr15-*deficient (*Gpr15*^−/−^) and *Gpr15*^+/+^ wild-type (WT) mice. Both groups were exposed to smoke for four weeks and then subjected to TNBS-induced CD-like and DSS-induced UC-like colitis models for comparison (Fig. 3a, b). Notably, compared with *Gpr15*^+/+^ mice subjected to the same TNBS induction, *Gpr15*^−/−^ mice presented alleviated symptoms of TNBS-induced colitis, including reduced body weight loss, a lower DAI, and colon shortening, as well as improved histopathology and colonoscopy scores (Fig. 3c, e, g and Supplementary Fig. 2a). Cigarette smoke exposure followed by TNBS-induced colitis led to reduced weight loss and diminished clinical scores, colon length, and less severe pathological and endoscopic findings in *Gpr15*^−/−^ mice than in *Gpr15*^+/+^ mice (Fig. 3c, e, g and Supplementary Fig. 2a). The inflammatory mediators *Il-1β*, *Il-6*, and *Tnf-α* were significantly reduced in *Gpr15*^−/−^ mice treated with TNBS, indicating a decreased inflammatory response (Fig. 3i). These reductions were also observed in *Gpr15*^−/−^ mice exposed to both cigarette smoke and TNBS-induced colitis compared with *Gpr15*^+/+^ mice (Fig. 3i).

In contrast, *Gpr15*^−/−^ mice treated with DSS presented greater weight loss, greater DAI scores, and colon shortening than did *Gpr15*^+/+^ mice (Fig. 3d and Supplementary Fig. 2c). Histological and endoscopic assessments revealed more severe inflammation in DSS-treated *Gpr15*^−/−^ mice, as indicated by increased cell infiltration, crypt distortion, goblet cell loss, and epithelial damage (Fig. 3f, h). Cigarette smoke exposure followed by DSS-induced colitis resulted in significantly greater weight loss, higher clinical scores, shorter colon lengths, and more severe pathological and endoscopic assessments in *Gpr15*^−/−^ mice than in *Gpr15*^+/+^ mice (Fig. 3d, f, h and Supplementary Fig. 2c). Compared with *Gpr15*^+/+^ mice, *Gpr15*^−/−^ mice exposed to DSS presented significantly higher levels of *Il-1β*, *Il-6*, and *Tnf-α* (Fig. 3j). Additionally, these cytokine levels were elevated in *Gpr15*^−/−^ mice exposed to cigarette smoke followed by DSS-induced colitis compared with those in *Gpr15*^+/+^ mice under the same conditions (Fig. 3j). After four weeks of cigarette smoke exposure, ELISA confirmed effective exposure, with significantly elevated urinary cotinine levels in the smoke-exposed group compared with those in the nonexposed group (Supplementary Fig. 2b, d). These results indicated that *Gpr15* deficiency alleviated the harmful effects of cigarette smoke on the progression of TNBS-induced colitis, effectively eliminating the protective effects of cigarette smoke on DSS-induced colitis.

Consistently, in *Gpr15*^+/+^ mice, prolonged cigarette smoke exposure in the TNBS model led to aggravated colitis, as reflected by greater weight loss, elevated DAI, colon shortening, and worsened histological and endoscopic scores, accompanied by increased *Gpr15* expression (Supplementary Fig. 3a, c, e, g). Correlation analysis revealed a significant association between *Gpr15* mRNA levels and disease severity (Supplementary Fig. 3i). Conversely, in the DSS model, extended smoke exposure alleviated colitis, while *Gpr15* expression also increased, underscoring model-dependent effects (Supplementary Fig. 3b, d, f, h, j). Collectively, these results highlight a strong association between *Gpr15* expression and colitis activity.

### Differential gene expression changes in the colonic tissue transcriptome of *Gpr15*-deficient mice with TNBS- and DSS-induced colitis under cigarette smoke exposure

**Fig. 4:**
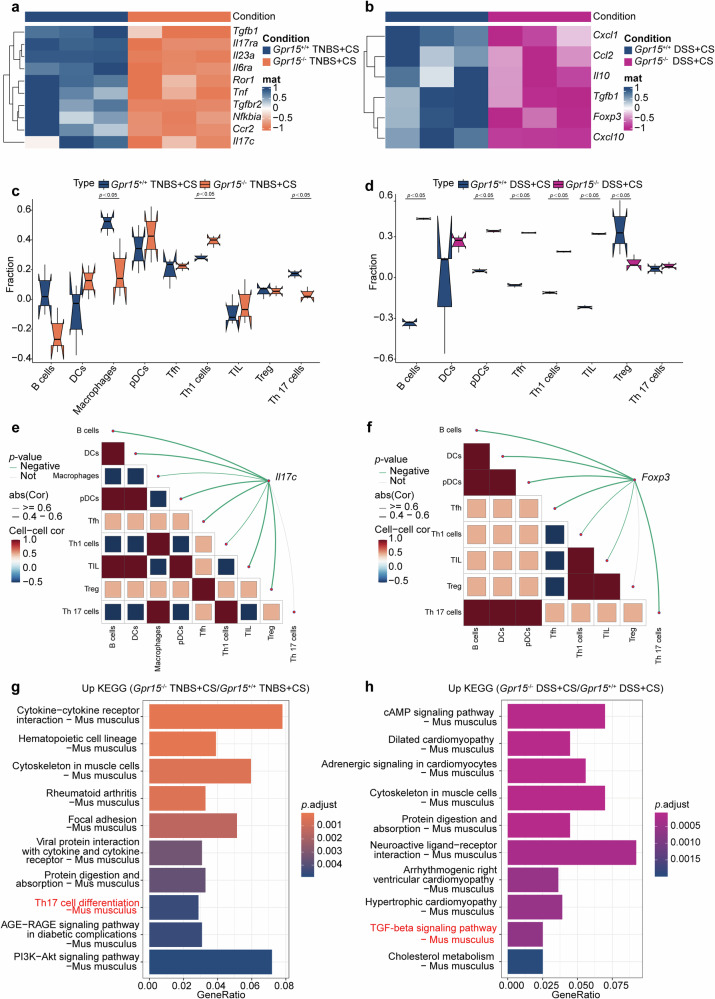
Effects of *Gpr15* deficiency on gene expression and immune cell infiltration in colitis with cigarette smoke exposure. **a** Heatmap of RNA sequencing data showing expression differences between *Gpr15*^−/−^ and *Gpr15*^+/+^ mice with TNBS-induced colitis under cigarette smoke exposure (*n* = 3). **b** Heatmap of RNA sequencing data showing expression differences between *Gpr15*^−/−^ and *Gpr15*^+/+^ mice with DSS-induced colitis under cigarette smoke exposure (*n* = 3). Each row represents a gene, and each column corresponds to a sample. Orange indicates decreased expression levels, whereas blue indicates increased expression levels relative to the mean. **c** Comparison of immune cell infiltration between *Gpr15*^−/−^ and *Gpr15*^+/+^ mice with TNBS-induced colitis under cigarette smoke exposure. **d** Comparison of immune cell infiltration between *Gpr15*^−/−^ and *Gpr15*^+/+^ mice with DSS-induced colitis under cigarette smoke exposure. The x-axis lists the cell types, whereas the y-axis depicts the fraction of each cell type. The blue dots represent samples from the *Gpr15*^+/+^ group, and the red dots represent samples from the *Gpr15*^−/−^ group. **e** Correlation of *Il17c* mRNA expression with immune cell infiltration in *Gpr15*^−/−^ and *Gpr15*^+/+^ mice during TNBS-induced colitis and **f** *Foxp3* mRNA expression with immune cell infiltration during DSS-induced colitis, both under cigarette smoke exposure. **g** KEGG pathway analysis revealed downregulation of the Th17 cell differentiation pathway in the colon of *Gpr15*^−/−^ mice during TNBS-induced colitis and **h** downregulation of the TGF-beta signaling pathway during DSS-induced colitis, both of which were induced by cigarette smoke exposure

To investigate the impact of *Gpr15* on gene expression in colonic tissue from mice with colitis under cigarette smoke exposure, RNA sequencing was performed on colonic tissue from both *Gpr15*^+/+^ and *Gpr15*^−/−^ mice. Heatmaps revealed that Th17-associated genes were significantly downregulated in the colonic tissue of TNBS-induced colitis *Gpr15*^−/−^ mice after cigarette smoke exposure (Fig. 4a). Surprisingly, *Tgfb1, Il17ra, Il23a, Il6ra, Ror1, Tnf, Tgfbr2, Ccr2*, and *Il17c* were downregulated by cigarette smoke exposure in TNBS-induced colitis *Gpr15*^−/−^ mice compared with similarly treated *Gpr15*^+/+^ mice (Fig. 4a). In DSS-induced colitis following cigarette smoke exposure, the six genes with the most significant differences (*Cxcl1, Ccl2, Il10, Tgfb1, Foxp3, and Cxcl10*), all of which are Treg signature genes, were consistently downregulated in *Gpr15*^−/−^ mice compared with those in *Gpr15*^+/+^ mice under identical experimental conditions (Fig. 4b). Quantitative analysis revealed that in *Gpr15*^−/−^ mice, cigarette smoke combined with TNBS reduced the Th17 cell ratio without affecting Treg responses, in contrast to the findings in *Gpr15*^+/+^ mice under the same conditions (Fig. 4c). Similarly, cigarette smoke combined with DSS in *Gpr15*^−/−^ mice led to a reduction in the Treg cell ratio without altering Th17 populations, differing from the findings in *Gpr15*^+/+^ mice exposed to the same treatment (Fig. 4d).

*Gpr15* deficiency significantly weakened the association between *Il17c* and immune cells, particularly Th17 cells, in the TNBS-induced colitis group following cigarette smoke exposure (Fig. 4e). Conversely, *Gpr15* deficiency in the DSS-induced colitis group under the same conditions significantly reduced the correlation with *Foxp3* and Treg cells (Fig. 4f). To identify the signaling pathways involved in the colitis model following cigarette smoke exposure, KEGG pathway enrichment analysis was performed (Fig. 4g–h). The Th17 signaling pathway was downregulated in TNBS-induced colitis *Gpr15*^−/−^ mice following cigarette smoke exposure (Fig. 4g). Similarly, the TGF-beta signaling pathway was also suppressed in DSS-induced colitis *Gpr15*^−/−^ mice under the same conditions (Fig. 4h). These results demonstrate that *Gpr15* deficiency attenuates the Th17 cell signaling pathway in TNBS-induced colitis and disrupts the Treg cell signaling pathway in DSS-induced colitis under both conditions of cigarette smoke exposure.

### Th17 cells exacerbate TNBS-induced colitis, whereas Treg cells alleviate DSS-induced colitis under smoke exposure

**Fig. 5:**
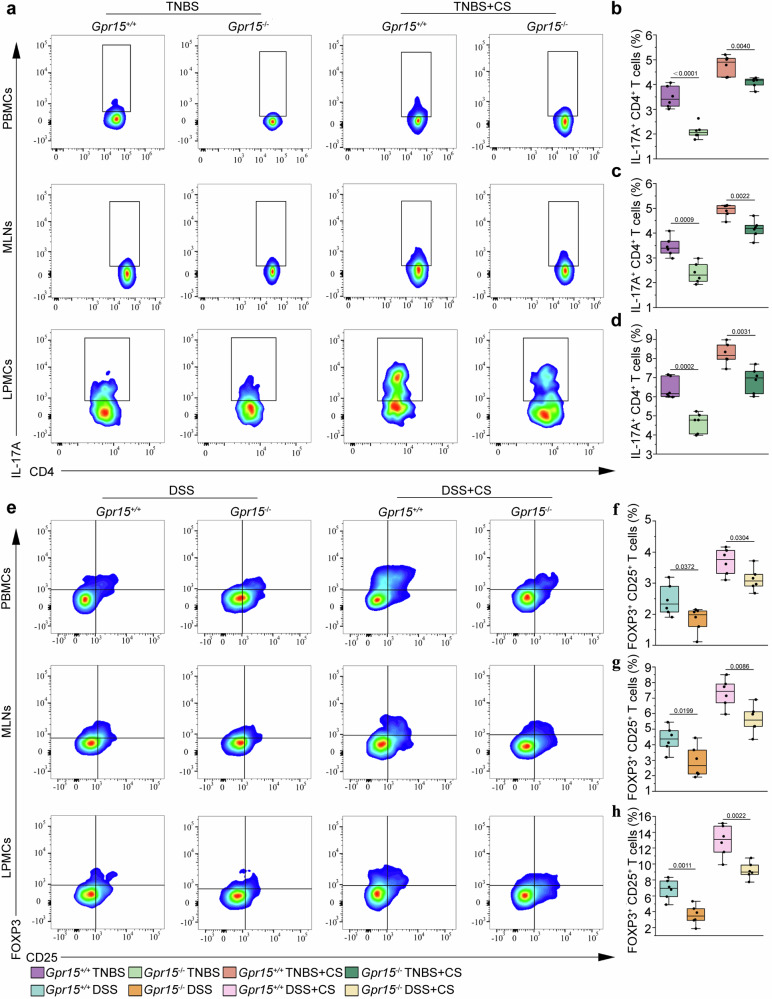
GPR15 modulates the severity of colitis in mice exposed to cigarette smoke: exacerbation of TNBS-induced colitis via Th17 cell activation and alleviation of DSS-induced colitis via Treg-mediated regulation. **a** Representative gating strategy for CD4^+^ IL-17A^+^ (Th17) cells in the peripheral blood mononuclear cells (PBMCs, top), mesenteric lymph node cells (MLNs, middle), and colonic lamina propria mononuclear cells (LPMCs, bottom) across different groups in the TNBS model under cigarette smoke exposure. The proportions of CD4^+^ IL-17A^+^ (Th17) cells in the PBMCs, (**b**, **c**) MLNs and (**d**) LPMCs. **e** Flow cytometry plots depicting gating strategies for CD25^+^ FOXP3^+^ (Treg) cells in the PBMCs (top), MLNs (middle), and LPMCs (bottom) across various cohorts in the DSS model under cigarette smoke exposure. The ratio of Treg cells within the PBMCs (**f**, **g**) MLNs, and **h** LPMCs was detected by flow cytometry and statistically analyzed. *n* = 6. The data are presented as the means ± SEMs; *p < 0.05, **p < 0.01, and ***p < 0.001 according to two-way ANOVA followed by multiple comparisons. All the experiments were repeated three times and yielded consistent results

To explore the role of *Gpr15* in Th17 cell regulation during TNBS-induced colitis following cigarette smoke exposure, we performed flow cytometric analysis of peripheral blood mononuclear cells (PBMCs), mesenteric lymph node cells (MLNs), and colonic lamina propria mononuclear cells (LPMCs) (Supplementary Figs. 4-6). In TNBS-induced *Gpr15*^−/−^ mice, the proportion of Th17 cells was significantly lower than that in TNBS-treated *Gpr15*^+/+^ mice (Fig. 5a–d). Both groups were exposed to cigarette smoke and TNBS, which resulted in a less pronounced Th17 reduction in *Gpr15*^−/−^ mice (Fig. 5a–d). Additionally, the percentage of Treg cells remained unchanged in both *Gpr15*^+/+^ and *Gpr15*^−/−^ mice subjected to smoke and TNBS-induced colitis (Supplementary Fig. 7a-d).

In DSS-induced colitis, flow cytometry revealed significantly fewer Treg cells among the mononuclear cells isolated from the PBMCs, MLNs, and LPMCs of *Gpr15*^−/−^ mice compared with those of *Gpr15*^+/+^ mice (Fig. 5e–h). In combination with DSS-induced colitis, *Gpr15*^−/−^ mice presented a greater reduction in the percentage of Treg cells than did *Gpr15*^+/+^ mice (Fig. 5e–h). No significant differences in Th17 cell percentages were detected between *Gpr15*^−/−^ and *Gpr15*^+/+^ mice under these conditions (Supplementary Fig. 7e-h). These results suggested that cigarette smoke exposure worsened TNBS-induced colitis by increasing the number of Th17 cells via GPR15, while it reduced the severity of DSS-induced colitis through Treg cells via GPR15.

### GPR15 enhances IL-17A expression in TNBS-induced colitis and upregulates FOXP3 expression in DSS-induced colitis, both of which occur following cigarette smoke exposure

**Fig. 6:**
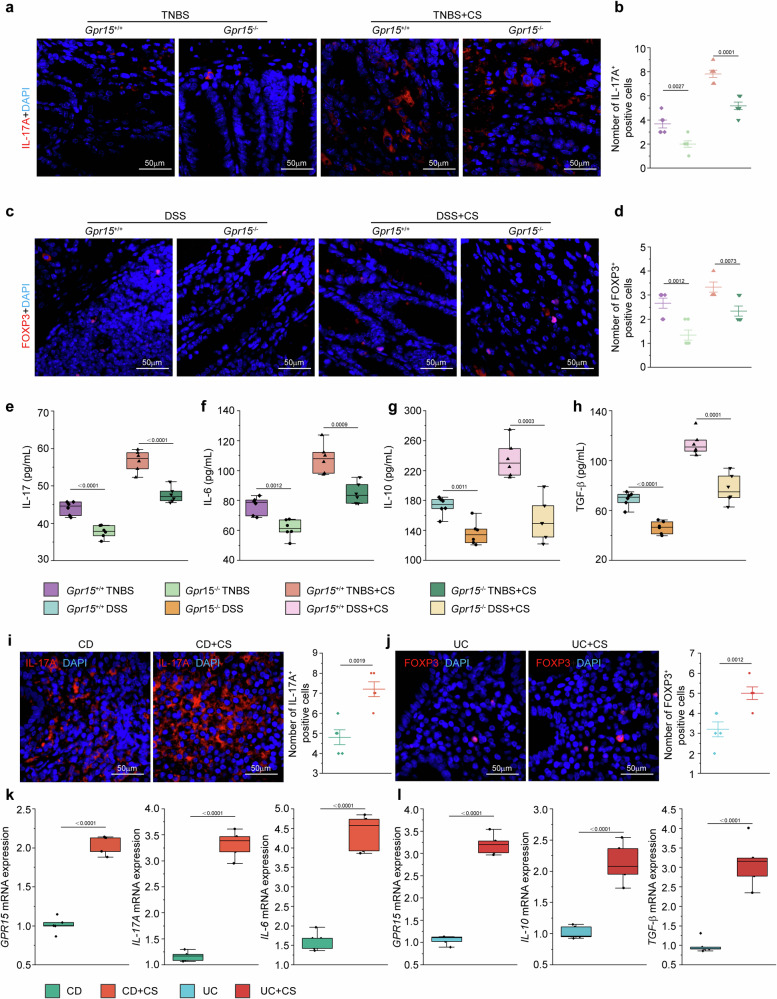
GPR15 drives Th17 cell responses in TNBS-induced colitis and maintains Treg functionality in DSS-induced colitis. **a** IL-17A^+^ cells detected by IF staining. IL-17A is shown in red, and nuclei are stained with DAPI (blue). **b** ImageJ analysis of the number of IL-17A^+^ cells in five randomly selected fields per group. **c** Representative IF images of FOXP3^+^ cells. FOXP3 (red) and nuclei (DAPI, blue). **d** Quantification of FOXP3^+^ cells in five random fields per group via ImageJ. **e** IL-17A and **f** IL-6 levels in colon tissue homogenates from *Gpr15*^+/+^ and *Gpr15*^−/−^ mice with TNBS-induced colitis with or without cigarette smoke exposure were measured via ELISA. **g** IL-10 and **h** TGF-β levels were similarly measured in *Gpr15*^+/+^ and *Gpr15*^−/−^ mice with DSS-induced colitis under the same conditions. **i** Representative IF images of IL-17A expression in CD patients with or without cigarette smoke exposure (IL-17A: red; nuclei: DAPI, blue), with quantification of IL-17A^+^ cells (*n* = 5). **j** Representative IF images of FOXP3 expression in colon tissues from UC patients under cigarette smoke exposure or nonexposure conditions. FOXP3 is shown in red, and nuclei are stained with DAPI (blue). The quantification of FOXP3^+^ cells is presented (*n* = 5). **k** mRNA expression levels of *GPR15*, *IL-17A*, and *IL-6* were analyzed in the CD group (*n* = 5). **l** *GPR15*, *IL-10*, and *TGF-β* levels were examined in the UC group (*n* = 5). Scale bar: 50 μm. The data are expressed as the means ± SEMs. Statistical significance was determined via two-way ANOVA with multiple comparisons, where *p < 0.05, **p < 0.01, and ***p < 0.001. All experiments were conducted in three biological replicates, yielding consistent results, with six mice included in each group for the animal studies

We evaluated IL-17A expression in TNBS-induced colonic inflammation induced by smoke exposure via immunofluorescence. IL-17A production and Th17 cell accumulation were significantly lower in the colons of *Gpr15*^−/−^ mice than in those of *Gpr15*^+/+^ mice with TNBS-induced colitis (Fig. 6a, b). Notably, Th17 cell accumulation was markedly lower in *Gpr15*^−/−^ mice exposed to TNBS colitis and cigarette smoke than in *Gpr15*^+/+^ mice under the same conditions (Fig. 6a, b). Similarly, in the DSS-induced colitis model, *Gpr15*^−/−^ mice presented significantly lower Foxp3 expression in the colon than did *Gpr15*^+/+^ mice (Fig. 6c, d). Immunofluorescence staining further revealed a substantial decrease in FOXP3-positive Treg cells in the colons of *Gpr15*^−/−^ mice compared with those in the colons of *Gpr15*^+/+^ mice under cigarette smoke exposure and DSS treatment (Fig. 6c, d).

ELISAs revealed significantly lower IL-17 and IL-6 levels in *Gpr15*^−/−^ mice with TNBS-induced colitis with or without smoke exposure (Fig. 6e, f). Compared with *Gpr15*^+/+^ mice, *Gpr15*^−/−^ mice presented reduced IL-10 and TGF-β levels, both with and without smoke exposure (Fig. 6g, h). No significant differences were found in the IL-10 or TGF-β levels in the TNBS group following smoke exposure or in the IL-17 or IL-6 levels in the DSS group. Furthermore, IL-4 and IFN-γ secretion did not differ between the TNBS and DSS groups (Supplementary Fig. 8a–h).

To assess IL-17A and FOXP3 expression in colonic tissue samples from IBD patients, immunofluorescence was used for visualization and quantitative analysis. In IBD patients, immunofluorescence revealed increased IL-17A^+^ cells in cigarette smoke-exposed CD patients and increased FOXP3^+^ cells in smoke-exposed UC patients (Fig. 6i, j). *GPR15*, *IL-17A*, and *IL-6* mRNA expression was increased in smoke-exposed CD patients, whereas *GPR15*, *IL-10*, and *TGF-β* mRNA expression was increased in smoke-exposed UC patients (Fig. 6k, l). *IL-4* and *IFN-γ* mRNA expression did not significantly differ between CD patients and UC patients with smoke exposure (Supplementary Fig. 8i–l).

To elucidate the functional impact of GPR15 on Th17 and Treg cells, adoptive transfer experiments were conducted. Th17 cells isolated from cigarette smoke-treated *Gpr15*^+/+^ and *Gpr15*^−/−^ mice were transferred into *Gpr15*^−/−^ mice with TNBS-induced colitis. The results indicated that Th17 cells from *Gpr15*^+/+^ mice significantly exacerbated inflammation, suggesting that smoking enhances Th17 pathogenicity via *Gpr15* (Supplementary Fig. 9a, c, e). Conversely, Treg cells from cigarette smoke-treated *Gpr15*^+/+^ and *Gpr15*^−/−^ mice were transferred into *Gpr15*^−/−^ mice with DSS-induced colitis to assess their protective effects. Treg cells from *Gpr15*^+/+^ mice conferred substantial protection, whereas those from *Gpr15*^−/−^ mice were ineffective, indicating that smoking enhances the suppressive function of Tregs through *Gpr15* (Supplementary Fig. 9b, d, f). These results suggest that GPR15 mediates distinct immune responses in colitis under cigarette smoke exposure, promoting Th17-driven inflammation in TNBS-induced colitis while supporting Treg-mediated regulation in DSS-induced colitis.

### Cigarette smoke extract promotes naïve CD4^+^ T-cell proliferation and differentiation by regulating GPR15 in vitro

**Fig. 7:**
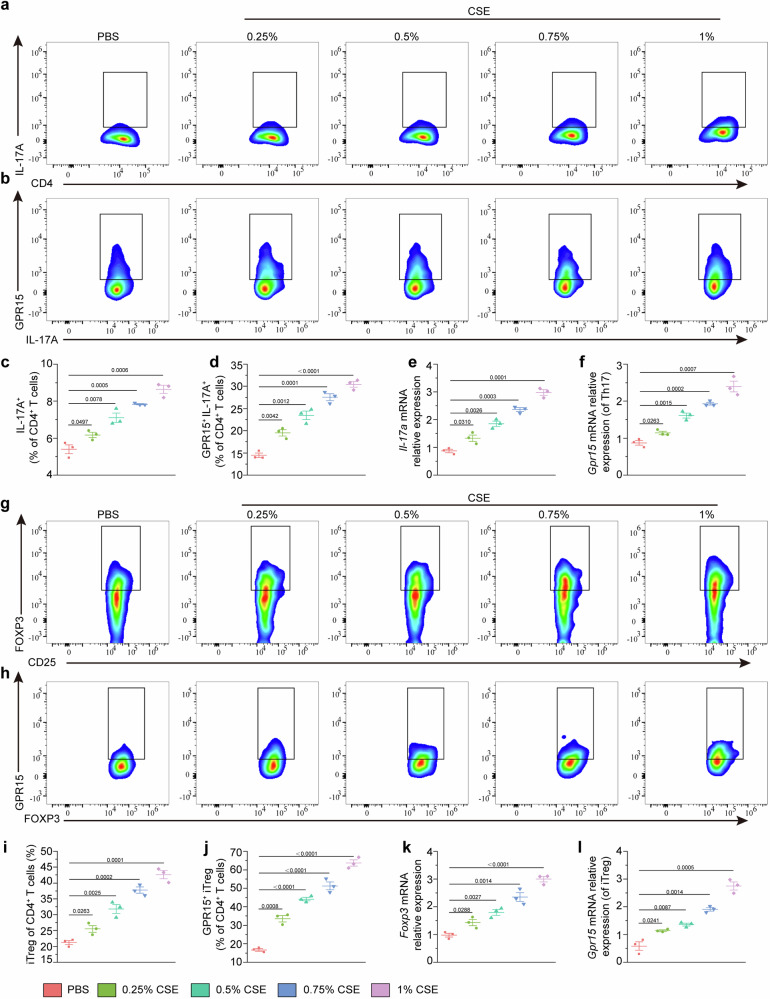
Exposure to CSE promotes the differentiation of naïve CD4^+^ T cells into Th17 and iTreg cells through GPR15 in vitro. **a** Representative gating strategy for CD4^+^ IL-17A^+^ (Th17) cells among stimulated naïve CD4^+^ T cells cultured with different concentrations of CSE. **b** Typical flow cytometry plots and gating for GPR15 expression in Th17 cells stimulated with graded concentrations of CSE. **c** Quantification of the Th17 cell frequency in stimulated naïve CD4^+^ T cells cultured with varying concentrations of CSE. **d** GPR15 expression increased in Th17 cells after stimulation with graded concentrations of CSE. **e** Relative mRNA expression of *Il-17a* and **f** *Gpr15* in CSE-treated Th17 cells. **g** Representative flow cytometry analysis showing the gating strategy for FOXP3^+^ CD25^+^ (iTreg) cells. **h** Flow cytometry analysis of GPR15 expression in iTreg cells cultured with various CSE concentrations. **i** iTreg frequencies across groups. **j** Comparison of GPR15 protein expression in iTreg cells cultured with or without different concentrations of CSE. **k** Relative mRNA expression of *Foxp3* and **l** *Gpr15* in CSE-treated iTreg cells. The data are presented as the means ± SEMs (*n* = 3); *p < 0.05, **p < 0.01, and ***p < 0.001 according to two-way ANOVA with multiple comparisons. All the experiments were performed in three biological replicates with consistent results

To explore the effect of cigarette smoke extract (CSE) on Th17 cell differentiation via GPR15, flow cytometric analyses were performed (Supplementary Fig. 10). The results of the CCK-8 assay revealed that CSE at concentrations ranging from 0.25–1% had no significant effect on cell viability after 24 h of treatment (data not shown). Flow cytometric analysis revealed that coculturing CSE (0.25–1%) with naïve CD4⁺ T cells isolated from the spleens of *Gpr15*^+/+^ mice resulted in a significant increase in the proportion of IL-17⁺ cells, indicating that CSE promotes the differentiation of naïve CD4⁺ T cells into Th17 cells (Fig. 7a, c). Interestingly, in Th17 cells differentiated from naïve CD4^+^ T cells under CSE stimulation, GPR15 expression was increased in a concentration-dependent manner (Fig. 7b, d). Additionally, we observed an increase in *Il-17a* and *Gpr15* mRNA expression in CSE-treated Th17 cells (Fig. 7e, f). Mechanistically, CSE exposure led to a time-dependent increase in STAT3 phosphorylation during Th17 differentiation of naïve CD4⁺ T cells isolated from the spleens of *Gpr15*^+/+^ mice (Supplementary Fig. 11a).

Next, we evaluated the effect of CSE on GPR15-mediated differentiation of iTreg cells from naïve CD4^+^ T cells via flow cytometry. The results indicated that CSE stimulation (0.25%-1%) significantly increased the proportion of iTreg cells in a dose-dependent manner (Fig. 7g, i). Unexpectedly, the expression of GPR15 in iTreg cells also increased with increasing concentrations of CSE (Fig. 7h, j). Furthermore, CSE treatment induced a concentration-dependent increase in *Foxp3* and *Gpr15* mRNA expression in iTreg cells (Fig. 7k, l). Notably, CSE exposure during iTreg differentiation induced a time-dependent increase in SMAD3 and STAT5 phosphorylation in naïve CD4⁺ T cells isolated from *Gpr15*^+/+^ mice (Supplementary Fig. 11b). In contrast, phosphorylation of STAT3, SMAD3, and STAT5 was not detected under the same conditions in naïve CD4⁺ T cells isolated from *Gpr15*^−/−^ mice, highlighting the essential role of GPR15 in CSE-induced activation of these signaling pathways during CD4⁺ T-cell differentiation (Supplementary Fig. 11a, b). In contrast, CSE stimulation of naïve CD4⁺ T cells from *Gpr15*^⁻/⁻^ mice failed to induce either Th17 or iTreg differentiation (Supplementary Fig. 12a–f). Coculturing naïve CD4^+^ T cells isolated from the spleens of *Gpr15*^+/+^ mice with CSE (0.25–1%) did not increase IFN-γ or IL-4 expression, indicating that CSE does not influence their differentiation into Th1 or Th2 cells (Supplementary Fig. 12g–l). These results demonstrated that CSE influences the differentiation of Th17 and iTreg cells by modulating GPR15.

### Cigarette smoke exacerbates TNBS-induced colitis but alleviates DSS-induced ulcerative colitis in C57BL/6JGpt-Tg (human *GPR15*) mice

**Fig. 8:**
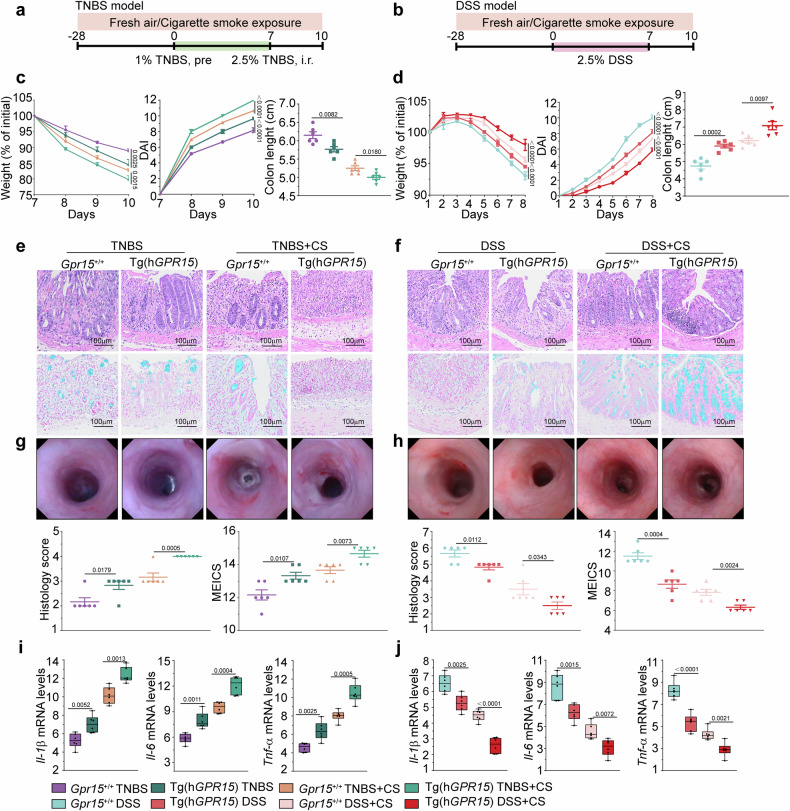
The dual role of cigarette smoke in colitis in C57BL/6JGpt-Tg (human *GPR15*) mice intensifies TNBS-induced colitis and mitigates DSS-induced colitis. **a** Experimental setup diagram for the Tg (human *GPR15*) mouse model subjected to cigarette smoke exposure and the TNBS-induced colitis model. **b** Diagram of the experimental framework for the Tg (human *GPR15*) mouse model exposed to cigarette smoke and the DSS-induced colitis model. **c** Body weight percentage, DAI scores, and colon length were recorded for the TNBS-induced colitis model. **d** Body weight percentage, DAI scores, and colon length were assessed in the DSS-induced colitis model. **e** Illustrative colon section images for the TNBS model: H&E staining is presented at the top left, Alcian blue staining at the bottom left, and the histology score on the left of the left panel. **f** Illustrative colon section images for the DSS model: H&E staining is presented at the top right, alcian blue staining at the bottom right, and the histology score at the bottom left of the right panel. **g** Left: TNBS group colonoscopy images and MEICS scores (bottom right of the left panel). **h** Right: DSS group colonoscopy images and MEICS scores (bottom right of the right panel). **i** The mRNA expression levels of *Il-1β*, *Il-6*, and *Tnf-α* in the TNBS model group are shown (left). **j** The mRNA expression levels of *Il-1β, Il-6*, and *Tnf-α* in the TNBS model group are shown (right). Six male mice were included in each group. The data are presented as the means ± SEMs; *p < 0.05, **p < 0.01, and *p < 0.001 according to two-way ANOVA with multiple comparisons. All the experiments were repeated three times and yielded consistent results

To further investigate the effects of GPR15 overexpression on smoking-related colitis, we induced acute colitis via TNBS and DSS in transgenic C57BL/6JGpt-Tg (human *GPR15*) mice and their littermate controls. We exposed these mice to cigarette smoke for four weeks, followed by 2.5% TNBS administration in the fifth week (Fig. 8a). In the acute phase, the TNBS-treated Tg(h*GPR15*) mice presented more severe intestinal inflammation than the *Gpr15*^+/+^ mice did (Fig. 8c, e, g and Supplementary Fig. 13a). Compared with *Gpr15*^+/+^ mice, Tg(h*GPR15*) mice exposed to TNBS and cigarette smoke presented reduced body weights, higher DAI scores, shorter colon lengths, and more severe colonic tissue damage (Fig. 8c, e and Supplementary Fig. 13a). Colonoscopy revealed marked redness and swelling of the colonic mucosa, along with multiple ulcers and bleeding points, indicating severe inflammation (Fig. 8g). Compared with *Gpr15*^+/+^ mice, Tg(h*GPR15*) mice treated with TNBS presented significantly higher levels of *Il-1β*, *Il-6*, and *Tnf-α* mRNA (Fig. 8i). Additionally, exposure to cigarette smoke and TNBS-induced colitis further elevated cytokine levels in Tg(h*GPR15*) mice compared with those in *Gpr15*^+/+^ mice (Fig. 8i).

Compared with those in *Gpr15*^+/+^ mice, DSS treatment resulted in milder colonic inflammation in Tg(h*GPR15*) mice, characterized by less weight loss, lower DAI scores, minimal colon shortening, and reduced histopathological and endoscopic scores (Fig. 8b, d, f, h and Supplementary Fig. 13c). Compared with the same conditions, combined cigarette smoke and DSS treatment significantly reduced body weight loss, DAI, and colon shortening and colonic tissue scores in Tg(hGPR15) mice compared with those in *Gpr15*^+/+^ mice (Fig. 8b, d, f and Supplementary Fig. 13c). Colonoscopy revealed fewer inflammatory changes in Tg(h*GPR15*) mice (Fig. 8h). Compared with *Gpr15*^+/+^ mice, DSS-treated Tg(h*GPR15*) mice presented significantly lower levels of *Il-1β*, *Il-6*, and *Tnf-α* mRNA (Fig. 8j). Compared with *Gpr15*^+/+^ mice, exposure to cigarette smoke followed by DSS-induced colitis further reduced the levels of these cytokines (Fig. 8j). Moreover, the urinary cotinine levels confirmed significant smoke uptake (Supplementary Fig. 13b, d).

Collectively, these results indicate that the Tg(h*GPR15*) phenotype with elevated GPR15 levels could aggravate TNBS-induced colitis while offering better protection against DSS-induced ulcerative colitis under cigarette smoke exposure.

### A schematic model illustrating how GPR15 differentially regulates the effects of cigarette smoke exposure on CD and UC

**Fig. 9:**
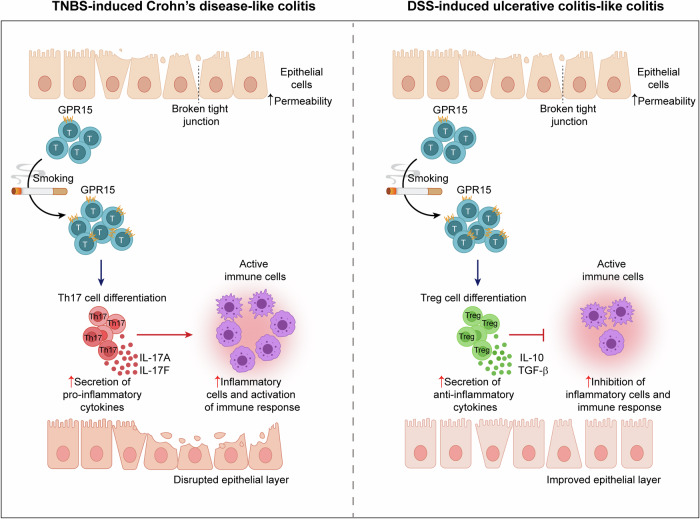
Mechanism of GPR15 action in IBD following cigarette smoke exposure. In TNBS-induced Crohn’s disease-like colitis, exposure to cigarette smoke causes the upregulation of GPR15 expression in T cells, which facilitates their differentiation into Th17 cells. These Th17 cells secrete proinflammatory cytokines, including IL-17A and IL-17F, which exacerbate the inflammatory response and worsen intestinal inflammation. Conversely, in DSS-induced ulcerative colitis-like colitis, cigarette smoke similarly increases GPR15 expression in T cells, leading to their differentiation into Treg cells. Treg cells produce anti-inflammatory cytokines such as IL-10 and TGF-β, which inhibit inflammatory processes and alleviate intestinal inflammatory conditions

In TNBS-induced CD-like colitis, cigarette smoke exposure upregulates *GPR15* expression on T cells, driving their differentiation into Th17 cells and exacerbating disease progression. In contrast, in DSS-induced UC-like colitis, cigarette smoke exposure similarly increases GPR15 expression on T cells but promotes their differentiation into Treg cells, conferring a protective effect against disease progression (Fig. 9).

## Discussion

To address the longstanding paradox of the divergent effects of cigarette smoke on CD and UC, this study integrated large-scale, population-based epidemiological analysis from the UK Biobank with comprehensive functional investigations in murine models of cigarette smoke-induced colitis. This integrative approach enabled a more precise delineation of the genetic regulator GPR15 in mediating distinct immunological responses to cigarette smoke across different IBD subtypes. These findings indicate that environmental exposure, such as exposure to cigarette smoke, modulates disease risk and progression through intricate interactions with genetic factors such as GPR15, thereby influencing disease susceptibility and phenotype. By bridging epidemiological evidence with mechanistic insights, this work advances our understanding of how cigarette smoke interacts with genetic regulators to affect immune responses and sheds new light on the complexity and heterogeneity underlying IBD pathogenesis.^23^

Importantly, our results identify GPR15 as a crucial mediator that transduces environmental signals from cigarette smoke to influence immune responses in specific IBD models. Specifically, prolonged exposure to cigarette smoke has contrasting, model-dependent effects on colitis severity, whereby upregulation of *Gpr15* expression is associated with exacerbated disease in the TNBS model but mitigated disease in the DSS model. Notably, cigarette smoke exposure induces pronounced and selective upregulation of GPR15 expression in colonic immune cells, whereas epithelial compartments exhibit minimal changes, underscoring a cell type-specific regulatory pattern (Supplementary Fig. 14a, b). Furthermore, the loss of *Gpr15* has divergent effects on chemically induced colitis under cigarette smoke exposure, conferring protection in TNBS-induced colitis while aggravating disease in the DSS model. These findings highlight the differential role of Gpr15 in regulating intestinal inflammation following smoke exposure. However, the incomplete loss or reversal of the effects of cigarette smoke in mice with *Gpr15* deficiency demonstrates that colitis severity is modulated by both Gpr15-dependent and Gpr15-independent mechanisms. For example, nicotine-mediated protection occurs independently of GPR15, suggesting the presence of parallel signaling pathways (Supplementary Fig. 15a–j). These findings highlight the complexity of smoke-associated intestinal inflammation and indicate that targeting GPR15 alone may be insufficient as a therapeutic strategy. Further investigations into additional molecular pathways, such as those involving the aryl hydrocarbon receptor, may yield new insights and therapeutic avenues for IBD.^24^

Building upon these findings, further mechanistic investigations highlight the context-dependent immunomodulatory role of GPR15 in shaping T-cell responses in models of colitis following cigarette smoke exposure. GPR15 regulates distinct immunological pathways under these conditions, including Th17 responses in TNBS-induced colitis and Treg responses in DSS-induced colitis. Mechanistic evidence specifically indicates that, in the TNBS-induced colitis model, GPR15 promotes STAT3-dependent Th17 activation and IL-17-mediated pathology. In contrast, in the DSS-induced colitis model, GPR15 activation enhances SMAD3/STAT5 signaling and promotes the differentiation of iTregs, thereby contributing to intestinal integrity. Consistent with these findings, adoptive transfer studies have demonstrated that cigarette smoke exposure selectively enhances the proinflammatory properties of Th17 cells and the immunosuppressive activity of Treg cells through a GPR15-dependent mechanism, underscoring GPR15 as a central regulator of divergent T-cell–mediated immune responses to environmental factors in colitis. Moreover, interventions with the cognate ligand C10orf99 (GPR15L) further support the context-dependent function of GPR15 (Supplementary Fig. 16a–j). Together, these findings position GPR15 at the interface of proinflammatory and regulatory T-cell responses and help explain why cigarette smoke has opposing effects on CD and UC. While previous studies have highlighted the significance of Th1 and Th2 subsets in IBD, the current findings show that neither cigarette smoke nor GPR15 modulation substantially affects these cell populations (Supplementary Fig. 17a–h).^25^ These insights broaden our understanding of IBD pathogenesis and suggest new targets for therapeutic intervention beyond conventional anti-inflammatory or immunosuppressive strategies.

Several important limitations of this study should be acknowledged. First, while the TNBS and DSS murine models are widely utilized, they only partially recapitulate the chronicity, heterogeneity, and tissue complexity of human IBD and may not capture all aspects of mucosal immunopathology.^26^ Second, murine smoking models cannot fully mimic the smoking exposure history of humans because of physiological differences and inherent limitations in exposure methods, concentrations, durations, and other experimental parameters.^27^ Third, interspecies differences in immune regulation, particularly between mouse and human GPR15 signaling networks, present challenges for the direct translation of these experimental findings into clinical practice.^28^ Fourth, while this study provides insight into GPR15-associated modulation of T-cell populations, the precise molecular mechanisms underlying GPR15-dependent immune regulation remain incompletely understood, especially regarding its effects on Treg-to-Th1 differentiation and Th17 cell stability in the context of cigarette smoke exposure, warranting further investigation. Taken together, these limitations highlight the necessity of stratifying IBD patients by smoking status when considering GPR15-targeted interventions and underscore the importance of future studies employing chronic or humanized models to validate and extend these findings.

Collectively, these findings advance the mechanistic understanding of T-cell–mediated immune regulation in IBD by elucidating how environmental exposures, particularly cigarette smoke, interact with host genetic factors such as GPR15 to drive heterogeneity in the disease phenotype and course. Targeting GPR15 or its downstream signaling pathways could lead to more precise treatment strategies adapted to specific disease subtypes and individual patient factors, such as smoking history. In light of these insights, the continued pursuit of rigorous clinical and mechanistic studies will be critical to validate these observations and translate them into safe and effective targeted therapies accounting for the heterogeneity observed in IBD.

## Materials and methods

### Population cohort study and statistical analysis

Population data were accessed from the UK Biobank, which is a large-scale prospective database containing detailed genetic, health, and lifestyle information from approximately 500,000 individual participants aged between 40 and 69 years at recruitment.^29^ Smoking status was classified according to established epidemiological standards into four categories: never smoked, ever smoked, previously smoked, and currently smoked.^30^ Never smoking refers to individuals who have never smoked or have smoked fewer than 100 cigarettes (or the equivalent amount of tobacco products) and are not currently smoking. Ever smoking includes those who have smoked at least 100 cigarettes (or equivalent), regardless of current status, thus encompassing both previous and current smokers. Previous smoking refers to those who have smoked at least 100 cigarettes but are currently not smoking, whereas current smoking consists of individuals who have smoked at least 100 cigarettes and continue to smoke at the time of assessment.

Hazard ratios (HRs) and their 95% confidence intervals (CIs) were calculated via Cox regression models to evaluate the associations between smoking status or history and incidence of inflammatory bowel disease (IBD). We adjusted our models for potential confounders, including age, body mass index (BMI), education score, sex, physical activity level, alcohol consumption state, processed meat intake, and genetic principal components. All the analyses were performed via R software (v4.1.3), and we adopted a two-sided testing strategy for all the statistical assessments, deeming the results to be significant at the stringent threshold of *p* < 0.05 (Supplementary Table 1).

### Whole-genome sequencing data processing and SNP filtering

Whole-genome sequencing (WGS) data from 490369 consented UK Biobank participants were analyzed via established protocols for sequencing, variant calling, and quality control.^31^ Population variant call format (pVCF) files from the Genome Analysis Toolkit (GATK) pipeline underwent further filtering, where genotype calls were masked if the genotype quality was <20, the read depth was <7 for single-nucleotide variants (SNVs) or <10 for insertions/deletions (indels), or if heterozygotes showed significant allelic imbalance (binomial *p* ≤ 0.001). Samples failing initial WGS quality control (*n* = 239) or having discordant phenotypic and genetic sex (*n* = 465; X-chromosome heterozygosity ≤0.30 as female, ≥0.80 as male) were excluded. Variants were retained if they met all the following criteria: allele alignment score ≥0.50, minor allele count (MAC) ≥ 1, missingness ≤0.5, heterozygous balance 0.175–0.825, homozygous balance ≥0.90, quality-by-depth ≥6, variant quality ≥10, and Hardy‒Weinberg equilibrium (HWE) *p* value ≥ 1 × 10^−100^, resulting in 1,159,259,784 high-confidence variants. Genetic ancestry was inferred via linkage disequilibrium-pruned autosomal single-nucleotide polymorphisms (SNPs; minor allele frequency [MAF] ≥0.01) projected onto the 1000 Genomes panel via KING software; 25,438 nonEuropean and 72,109 related (third-degree or closer) individuals were excluded.^32^ After merging the phenotypic data, 304,346 unrelated participants of European ancestry remained; analyses focused on 8746 SNPs (MAF ≥ 0.01) within 10 kilobases (kb) of *GPR15* (Supplementary Table 2).

### Adjustment for GPR15 locus genetic variation in statistical analyses

To control for confounding genetic variation at the GPR15 locus, 8,746 flanking markers were assessed for associations with Crohn’s disease (CD) and ulcerative colitis (UC) via generalized linear mixed models adjusted for age, sex, an age‒sex interaction, and the first ten principal components.^33^ Markers with *p* < 0.05 were LD-pruned (r² < 0.2), resulting in 24 and 2 independent variants for CD and UC, respectively. These variants and their interactions with smoking were included in Cox models via forward selection, retaining terms with *p* < 0.05 (Supplementary Table 2).

### Population study

Between September 2023 and September 2024, all patients with UC or CD evaluated by the Division of Gastroenterology at West China Hospital, either in outpatient clinics or as inpatients, were invited to participate in the study. Disease activity at enrollment was assessed via the Crohn’s Disease Activity Index (CDAI) for CD and the Mayo score for UC, with a flare defined as a CDAI > 150 for CD or a Mayo score > 2 (or partial Mayo score ≥ 2) for UC.^34^ The study adhered to the Declaration of Helsinki and was approved by the Ethics Committee of West China Hospital, Sichuan University, Chengdu, China (No. 2023[1081], [2024(500)]). Written informed consent was obtained from all participants. The clinical and experimental characteristics of the IBD patients, including their smoking history, are summarized in Supplementary Table 3.

### Animals

All the mice used were on a C57BL/6 background. Male wild-type C57BL/6 mice (*Gpr15*^+/+^) and *Gpr15*-deficient mice (*Gpr15*^−/−^) were obtained from Cyagen Biosciences (Suzhou, China). *Gpr15*-deficient mice were generated through embryonic stem cell targeting, and gene disruption was verified via quantitative PCR (qPCR) (Supplementary Table 4). The comprehensive methodologies and validation results are detailed in Supplementary Fig. 18. C57BL/6JGpt-Tg (human *GRP15*) transgenic mice were generated by GemPharmatech Co., Ltd. (Chengdu, China) via bacterial artificial chromosome (BAC) technology, ensuring the expression of the human *GPR15* sequence in the resulting individuals (Supplementary Fig. 19 and Supplementary Table 5). All the animals, aged 6 to 8 weeks, were maintained in a specific pathogen-free (SPF) facility, housed in plastic cages with five mice per cage, and provided with standard laboratory chow and water ad libitum. The environment was controlled with a 12-hour light/dark cycle. All procedures received approval from the Animal Care and Use Committee of West China Hospital, Sichuan University (ID: 20220214011).

### Cigarette smoke exposure

For this study, standard filtered cigarettes containing 11 mg of tar and 1 mg of nicotine sourced from the local market were used. The mice were exposed to whole-body cigarette smoke in a Plexiglas chamber (50 × 60 × 70 cm) connected to a smoking machine (CSM-I, Chengdu Ruituo Technology Co., Ltd.). The exposure protocol followed the International Organization for Standardization Protocol with a 35 mL puff/minute rate, lasting approximately 8 minutes per cigarette, and a 5-minute fresh air ventilation interval between cigarettes. Medical-grade air flowed at 3 L/min to maintain oxygen levels between 18% and 20%. Gravimetric analysis was performed via A/E glass fiber filters (PALL Life Sciences, Tijuana, Mexico) to measure the concentration of total particulate matter (TPM), which was approximately 200 mg/m³ within a chamber containing six cigarettes. The mice were exposed to smoke from six cigarettes twice daily, five days a week, for four weeks, whereas the control mice received only fresh air.

### TNBS-induced colitis in mice

TNBS-induced colitis was induced following established protocols, where acute colitis was induced by presensitizing mice with 150 μL of a 1% (w/v) TNBS solution (p2297, Sigma, Darmstadt, Germany) prepared in a 4:1 acetone-to-olive oil mixture and applied to a 1.5 × 1.5 cm shaved dorsal area between the shoulders.^35^ On day 8, after the mice were weighed and anesthetized, a 100 μL dose of 2.5% (w/v) TNBS in 50% ethanol was administered intrarectally via a catheter inserted 4 cm into the colon, with the mice maintained in a head-down position for 5 min. The control mice underwent the same procedures and received 45% ethanol in phosphate-buffered saline (PBS). The mice were monitored daily for survival, body weight, rectal bleeding, and stool consistency. Inflammation severity was assessed via established scoring criteria.

### DSS-induced colitis in mice

Acute colitis was induced via the oral administration of 2.5% (w/v) dextran sodium sulfate (DSS; MP Biomedicals, UK) in the drinking water for the indicated period, after which the water containing DSS was replaced with normal drinking water.^36^ The control animals were administered distilled water. Colitis severity was evaluated daily by monitoring body weight, diarrhea, and bloody stool occurrence. Stool samples were collected and assessed for occult blood via hemoccult blood test strips from Nanjing Jiancheng Bioengineering Institute (Nanjing, China) according to the manufacturer’s instructions. Colon inflammation was evaluated according to standardized scoring criteria. During the designated time period, the mice were euthanized, and colon tissue was obtained by trimming at the ileocecal junction and distal end of the rectum, followed by measurement of total length. The disease activity index (DAI) was calculated as a composite score on the basis of relative body weight loss, changes in the colon length/weight ratio, and the presence of occult blood in stool samples.^37^

### Colonoscopy

Colonoscopy was performed on experimental mice via a flexible video mini-endoscope (URF-V3; Olympus) under anesthesia with 1.5% to 2.0% isoflurane. The colon was gently insufflated with air via the working channel to visualize up to 3 cm of the proximal region and ensure sufficient distention. White light imaging was employed to examine the colon and identify lesions. Colitis severity was assessed blindly via the murine endoscopic index of colitis severity (MEICS).^38^

### Measurement of urine cotinine levels

Urine cotinine levels from cigarette smoke exposure were evaluated after the final exposure on day 28 via an enzyme-linked immunosorbent assay (ELISA) kit (Calbiotech, Spring Valley, CA, USA). Samples from at least six mice per group were analyzed in each experiment.

### Enzyme-linked immunosorbent assay

Cytokines, specifically IL-10 (CAT# RX203075M), IL-17A (CAT# RX203067M), IL-6 (CAT# RX2023049M), TGF-β (CAT# RX202402M), IL-4 (CAT# RX203051M), and INF-γ (CAT# RX203097M), were quantified in colon tissue homogenates via ELISA kits from Quanzhou Ruixinbio Biotechnology Co., Ltd. (Quanzhou, China) according to the manufacturer’s instructions. Capture antibodies (1:200) were used to coat the plate overnight at 4 °C. After blocking, the samples were incubated for 2 h at room temperature, followed by incubation with detection antibodies (1:200) for 1 h. Subsequently, streptavidin conjugated with streptavidin conjugated with horseradish peroxidase was added, and the substrate was added after 30 min. Finally, the absorbance value was detected via a microplate reader. The concentrations of the cytokines were obtained according to standard curves.

### Histological examination of colon tissue

The distal segments of colon tissue were collected and fixed overnight in 4% paraformaldehyde at 4 °C. After fixation, the samples were dehydrated with graded alcohol, cleared in xylene, embedded in paraffin, and sectioned into 3 µm slices. Following dewaxing, the sections were stained with H&E or alcian blue (Solarbio, Beijing, China) in accordance with the manufacturer’s protocols. Digital scanning and analysis of histopathology slides were conducted via NDP.viewer 2.0 software from Hamamatsu. Histopathological evaluation was conducted by blinded pathologists, who assigned scores on the basis of established parameters, including inflammatory cell infiltration, tissue destruction, crypt architectural distortion, and regenerative responses.^39^

### Immunofluorescence staining

For immunofluorescence staining, colons from the various groups of mice were cut into 3 μm sections via a freezing microtome. The sections were incubated for 1 h in buffer containing 5% normal blocking serum. The samples were subsequently stained with anti-IL-17A (eBioscience, 14-6988-82) and anti-FOXP3 (GeneTex, GTX107737) antibodies. Nuclear staining was performed using DAPI (Abcam, ab104139). Images were captured with an Olympus BX600 microscope equipped with a SPOT Flex camera (Olympus Corporation, Tokyo, Japan) and subsequently processed and analyzed via ImageJ software (version 1.53 m).

### Real-time fluorescent quantitative polymerase chain reaction

RNA was extracted from cultured cells and colon tissue via TRIzol reagent (Thermo Fisher Scientific, USA) according to the manufacturer’s protocol. RNA quality was evaluated with a NanoDrop 2000 (Thermo Fisher Scientific). First-strand cDNA synthesis was performed via a reverse transcription kit (Vazyme, Nanjing, China). Real-time fluorescent quantitative polymerase chain reaction (RT‒qPCR) was conducted with gene-specific primers (Qing Ke Bio; Supplementary Table 6) and SYBR Green PCR Master Mix (Takara Bio; RR820) according to the manufacturer’s instructions. Triplicate reactions were run on a LightCycler 96 PCR system (Roche). mRNA expression was normalized to that of β-actin, and the data were analyzed via the 2^−ΔΔCt^ method.

### Lymphocyte isolation

Peripheral blood mononuclear cells (PBMCs) were isolated from whole blood collected in K_2_ EDTA tubes (BD, Franklin Lakes, NJ) via density-gradient centrifugation via Ficoll‒Paque (GE Healthcare, cat. 17–1440–03). Following centrifugation at 500 × *g* for 20 min at 21 °C, the buffy coat was collected, washed twice with PBS, centrifuged again, and prepared for flow cytometry staining. Moreover, mesenteric lymph nodes (MLNs) from sacrificed mice were processed through 70 μm cell strainers, washed with PBS, and centrifuged at 500 × *g* and 4 °C for 5 min. The MLN cells were subsequently prepared for flow cytometry analysis.

To prepare lamina propria mononuclear cells (LPMCs), colons from TNBS- or DSS-treated mice were harvested and processed via established methods.^40^ The colons were excised, opened lengthwise, rinsed twice with cold PBS, and cut into 0.5 cm pieces. These pieces were incubated in Ca/Mg-free Hank’s balanced salt solution (HBSS, Sigma‒Aldrich) containing 30 mM ethylenediaminetetraacetic acid (EDTA, Sigma‒Aldrich) and 1.5 mM dithiothreitol (DTT, Sigma‒Aldrich) at 37 °C with shaking for three cycles of 10 min each. To isolate LPMCs, the tissues were digested in RPMI (Gibco) supplemented with 20% fetal bovine serum (FBS), 1 mg/mL collagenase IV, and 0.05 mg/mL DNase I at 37 °C for 30 min. The digested samples were then passed through 40 μm strainers, rinsed with PBS, and resuspended in 4 mL of 40% Percoll (Sigma‒Aldrich). This suspension was carefully layered onto 80% Percoll, and the mononuclear cells at the 40%-80% interface were collected following centrifugation at 500 × g and 4 °C for 8 minutes. Finally, LPMCs were resuspended in FACS buffer for further analysis.

### Flow cytometry analysis

For surface staining, the cells were incubated with antibodies, including anti-CD3-AF700, anti-CD4-FITC, anti-CD45-BV510, anti-CD8-Percp-cy5.5, anti-GPR15-APC, and anti-CD25-BV650 (all from eBioscience), for 30 min at 4 °C in the dark. Following incubation, the cells were washed and stimulated with 50 ng/mL phorbol 12-myristate 13-acetate (PMA), 750 ng/mL ionomycin, and 10 μg/mL brefeldin A (all from eBioscience) for 4 h at 37 °C in a 5% CO_2_ environment. For intracellular staining, the cells were subsequently harvested, washed with PBS, fixed, and permeabilized with a FOXP3/transcription factor fixation/permeabilization kit (Thermo Fisher Scientific). The cells were then stained with anti-IFN-γ-PE-Cy7, anti-IL-17-BV421, anti-IL-4-BV711, and anti-FOXP3-PE (all from eBioscience) for 40 min at 4 °C in the dark. The stained samples were then assessed via a Cytek Aurora and analyzed with FlowJo 10.8.1 software.

### Cell culture

Caco-2 epithelial colon cells were obtained from the American Type Culture Collection (ATCC, Manassas, VA, USA) and expanded in Dulbecco’s modified Eagle’s medium (DMEM) supplemented with 20% FBS (Gibco, Waltham, MA, USA) and 1% penicillin/streptomycin (Thermo Fisher Scientific, Waltham, MA, USA) to promote optimal growth and viability. For the experimental procedures, Caco-2 cells were subcultured and utilized at passages 5–10 in DMEM containing 10% FBS and 1% penicillin/streptomycin and incubated at 37 °C in a humidified atmosphere with 5% CO₂. Human colonic epithelial cells (HCoEpiC) were obtained from CliniSciences (CliniSciences, Guidonia Montecelio, Italy) and cultivated in colonic epithelial cell medium (HCoEpiCM) supplemented with 10% (v/v) colonic epithelial cell growth supplement (HCoEpiCGS; Zenbio, Durham, NC, USA) and 1% penicillin/streptomycin. HCoEpiC cells were maintained at 37 °C in 5% CO₂ and used for experiments between passages 5 and 8.

### In vitro Th17 and iTreg cell differentiation

Naïve CD4^+^ T cells were purified from splenocytes via the Mouse Naïve CD4^+^ T-cell Isolation Kit (CAT#19765; STEMCELL Technologies) strictly according to the manufacturer’s instructions and further sorted via a FACSAria II (BD Biosciences). Naïve CD4^+^ T cells (0.4 × 10^6^) were cultured in RPMI-1640 medium supplemented with 10% FBS (Gibco, CA, USA), 2 mM glutamine, 1% penicillin streptomycin (Gibco, CA, USA), 10 mmol/L N-2-hydroxyethylpiperazine-N’-2-ethanesulfonic acid (HEPES, Gibco, CA, USA), and 55 mmol/L 2-mercaptoethanol (Gibco, CA, USA) in 24-well plates. For iTreg induction, naïve T cells were stimulated with anti-CD3 (5 μg/mL)/anti-CD28 (5 μg/mL) plus IL-2 (2 ng/mL) in the presence of TGF-β (5 ng/mL) at 37 °C for the indicated times. To induce Th17 cell differentiation, the cells were treated with TGF-β (3 ng/mL), IL-6 (20 ng/mL), IL-23 (25 ng/mL), an anti-IL-4 antibody (5 μg/mL), and an anti-IFN-γ antibody (Pepro Tech). Splenocytes (1 × 10^6^ cells) were cultured with a specific concentration of cigarette smoke extract (CSE) for 24 h. Then, the expression of CD4, CD25, FOXP3, IL-17, and GPR15 in splenocytes was detected by flow cytometry.

### Preparation of cigarette smoke extract

CSE was prepared by combusting one standard filtered cigarette and channeling the smoke through 10 mL of PBS via a vacuum pump and a custom syringe-based apparatus to produce 100% CSE.^41^ The CSE solution was filtered through a 0.2 μm membrane to remove particulates. The 100% CSE solution was subsequently diluted with PBS to the required concentration (0.25%, 0.5%, 0.75%, or 1%).

### Adoptive transfer experiments

T-cell adoptive transfer was conducted as previously described.^42^ To investigate the role of Gpr15 in the effects of cigarette smoke exposure on immune function, Th17 and Treg cells were isolated from *Gpr15*^+/+^ and *Gpr15*^−/−^ mice exposed to cigarette smoke. After four weeks of cigarette smoke exposure, the spleens were harvested and ground in physiological saline to prepare single-cell suspensions, which were subsequently filtered. Flow cytometry was used to isolate and sort Th17 (CD4^+^IL-17A^+^) and Treg (CD4^+^CD25^+^FOXP3^+^) cells on the basis of specific surface markers. For the TNBS-induced colitis model, approximately 5 × 10^6^ Th17 cells from cigarette-exposed *Gpr15*^+/+^ and *Gpr15*^−/−^ mice were transferred into *Gpr15*^−/−^ mice via tail vein injection on the third day of colitis induction, and the clinical signs of colitis were monitored. In the DSS-induced colitis model, 2.5% DSS was administered in the drinking water to *Gpr15*^−/−^ mice to induce colitis, and on the third day of induction, approximately 5× 10^6^ Treg cells from cigarette-exposed *Gpr15*^+/+^ and *Gpr15*^−/−^ mice were transferred into *Gpr15*^−/−^ mice via tail vein injection.

### Western blot

The cells were lysed in RIPA buffer containing SIGMAFAST Protease Inhibitor (Cat. No. P8340) and Sigma Phosphatase Inhibitor (Cat. No. P5726) from Sigma‒Aldrich, in accordance with previously established protocols.^43^ Nitrocellulose membranes were incubated with primary antibodies against pSTAT3 (Tyr705, Cat: 9131), STAT3 (Cat: 4904), pSTAT5 (Tyr694, Cat: 9351), STAT5 (Cat: 94205), pSMAD3 (Ser423/425, Cat: 9520), and SMAD3 (Cat: 9523) from Cell Signaling Technology. Following incubation, the membranes were washed, and densitometric analysis was conducted via ImageJ software (NIH) to quantify the intensity of the protein bands.

### mRNA sequencing and bioinformatic analysis

Total RNA was extracted from mouse colon tissue via the peqGOLD Total RNA Kit (VWR International) according to the manufacturer’s instructions. The RNA purity and concentration were assessed with a NanoDrop spectrophotometer, a Qubit RNA High Sensitivity Assay, and an Agilent 2100 Bioanalyzer. Libraries were generated from 1 μg of total RNA per sample via the NEBNext Ultra RNA Library Prep Kit (New England Biolabs) and then indexed and amplified with Phusion High-Fidelity DNA Polymerase. Library quality was verified before sequencing with paired-end reads on an Illumina platform. The raw reads were quality filtered and processed to remove adapters, ambiguous nucleotides, and low-quality sequences. Only high-quality reads, as measured by the Q20, Q30, and GC content metrics, were retained for downstream analyses. Reads were mapped to the mouse reference genome via HISAT2, and gene-level counts were generated with featureCounts.

For differential expression analysis, gene-level count data were analyzed with DESeq2 (version 1.38.3) in R (version 4.2.3). To control for false positives resulting from multiple hypothesis tests inherent to RNA-seq experiments, *p*-values were adjusted via the Benjamini–Hochberg procedure to control the false discovery rate (FDR). Genes were identified as differentially expressed if they met both an FDR-adjusted *p*-value < 0.05 and an absolute log2-fold change > 1. Functional enrichment for biological processes and signaling pathways was performed via Kyoto Encyclopedia of Genes and Genomes (KEGG) pathway annotations with the clusterProfiler package (version 4.6.2) in R, which utilizes gene sets from MSigDB.

Given the pivotal role of immune mechanisms in IBD pathogenesis, immune cell infiltration was profiled with the CIBERSORT algorithm, enabling the quantification of 22 distinct immune cell subtypes and the comprehensive characterization of immune landscapes.^44^ Reference genome and gene model annotation files were sourced from genome browser platforms such as NCBI, UCSC, or Ensembl.

### Statistical analysis

All the results, excluding the RNA-seq data processed with R (version 4.1.2, R Foundation, Vienna, Austria), are reported as the means ± standard errors of the means (SEMs). Statistical analyses were conducted via GraphPad Prism 9.0 (GraphPad Software, San Diego, USA). For two-group comparisons, a *t*-test was employed. When more than two groups were compared, one-way ANOVA followed by Tukey’s multiple comparison test was used; significance was set at an adjusted *p*-value of < 0.05. Flow cytometry data were analyzed via FlowJo 10.8.1 software (Tree Star, Inc., Ashland, USA).

## Supplementary information

Supplementary Materials

## Data Availability

All the data required to support the conclusions of this study are provided in the main text and Supplementary Materials. The raw transcriptome data have been deposited in the Genome Sequence Archive (GSA) under project number CRA027654 (mouse tissue) and can be accessed at https://ngdc.cncb.ac.cn/gsa/search?searchTerm=CRA027654.
