# Supplementary material for: GPR15 differentially regulates the effects of cigarette smoke exposure on Crohn’s disease and ulcerative colitis
Source: Signal Transduct Target Ther. 2025 Sep 17;10:306. doi: 10.1038/s41392-025-02384-8 (PMC12441133; doi:10.1038/s41392-025-02384-8)
Supplement: Supplementary file 1 — Supplementary Materials [file 41392_2025_2384_MOESM1_ESM.docx]

Supplementary Materials for

**GPR15 differentially regulates the effects of cigarette smoke exposure on Crohn’s disease and ulcerative colitis**

**Authors:** Luhua Gao^1,*^, Huaping Zheng^1,*^, Qing Zhao^1,*^, Yubin Wang^1,*^, Yong He^2,*^, Can Hou^3,4,*^, Na Yang^5^, Kun Liang^6^, Wenjian Meng^7^, Xuefei He^1^, Kun Zheng^1^, Wenning Tian^1^, Jiacheng Zhang^1^, Ting Zhang^1^, Hui Mao^1^, Liming Zhang^8^, Jingyu Zhang^9^, Jingqiu Cheng^1^, Jürgen Brosius^1^, Huan Song ^3,4,†^, Yuchuan Huang^6,†^, Yi Chen^7,†^, Cheng Deng^1,10,†^

^*^These authors contributed equally to this work.

Corresponding author(s): [dengcheng@wchscu.cn](mailto:dengcheng@wchscu.cn) (C.D.); toddy@scu.edu.cn (Y.C.); [yuchuanghuang@163.com](mailto:yuchuanghuang@163.com) (Y.H.); [songhuan@wchscu.cn](mailto:songhuan@wchscu.cn) (H.S.)

**This PDF file includes:**

Supplementary Figures S1 to S19

Supplementary Tables S1 to S6

**Supplementary Figure 1 a, c** Colon tissue was harvested on the final day of the experiment. Representative images of colon tissues from each group are displayed. Scale bar: 1 cm. **b, d** Cotinine measurements in murine urine. The data are presented as the means ± SEMs (n=6). Statistical significance was evaluated using two-way ANOVA with multiple comparisons (**p* < 0.05, ***p* < 0.01, ****p* < 0.001). All the experiments were conducted in three biological replicates with reproducible results.

**Supplementary Figure 2** **a, c** Colon tissue was collected at the conclusion of the experiment. Displayed are representative images from each group. Scale bar: 1 cm. **b, d** Urinary cotinine concentrations in mice. The data are presented as the means ± SEMs (n=6). Two-way ANOVA with multiple comparisons was used to assess significance (**p* < 0.05, ***p* < 0.01, ****p* < 0.001). Experiments were performed in three biological replicates with consistent results.

**Supplementary Figure 3 Prolonged exposure to cigarette smoke in TNBS and DSS colitis models significantly enhances *Gpr1*5 expression, correlating with disease severity**

**a** The experimental timeline of the TNBS model delineates the periods of exposure to either fresh air or cigarette smoke. **b** The experimental timeline of the DSS model outlines the specific periods of exposure to either fresh air or cigarette smoke. **c** Percentage alterations in body weight, DAI scores, and colon length in the TNBS colitis model. **d** Percentage alterations in body weight, DAI scores, and colon length in the DSS colitis model. **e** Left: Representative histological images of colon sections from the TNBS model stained with H&E; Histological scores reflecting the degree of tissue damage. **f** Right: Histological images of colon sections in the DSS model, stained with H&E; Histological scores reflecting the degree of tissue damage. **g** Colonoscopy images from the TNBS group are shown on the left with the corresponding MEICS. **h** Colonoscopy images from the DSS group are shown on the right with the corresponding MEICS. **i** *Gpr15* mRNA expression levels in the TNBS group are presented, along with correlation plots illustrating the relationships between *Gpr15* expression and clinical parameters, including the DAI, histological score, and MEICS. **j** *Gpr15* mRNA expression levels in the DSS group are presented, along with correlation plots illustrating the relationships between *Gpr15* expression and clinical parameters, including the DAI, histological score, and MEICS. Correlation coefficients (cor) and corresponding *p*-values underscore the statistical significance of these associations. Scale bar, 100 μm. Each group consisted of six male mice. The data are presented as the means ± SEMs (n=6); **p* < 0.05, ***p* < 0.01, ****p* < 0.001 and ns (not significant) determined by two-way ANOVA with multiple comparisons. All the experiments were conducted independently three times as biological replicates, yielding consistent results.

**Supplementary Figure 4 corresponds to the flow cytometry gating strategy for peripheral blood mononuclear cells described in Fig. 5, as well as in Supplementary Figures 7 and 17**

The flow cytometry gating strategy was employed to delineate CD4^+^ and CD8^+^ T cell subsets, including CD4^+^ IL-4^+^, CD25^+^ FOXP3^+^, CD4^+^ IFN-γ^+^, and CD4^+^ IL-17A^+^ populations, within peripheral blood mononuclear cells (PBMCs) isolated from the blood of mice exposed to cigarette smoke and those subjected to TNBS or DSS-induced colitis.

**Supplementary Figure 5 demonstrates the flow cytometry gating strategy utilized for mesenteric lymph nodes cells, as comprehensively detailed in Fig. 5 and Supplementary Figures 7 and 17**

Flow cytometry gating was used to analyze CD4^+^ and CD8^+^ T cell subsets, such as CD4^+^ IL-4^+^, CD25^+^ FOXP3^+^, CD4^+^ IFN-γ^+^, and CD4^+^ IL-17A^+^, in mesenteric lymph node cells (MLNs) from mice exposed to cigarette smoke or colitis induced by TNBS or DSS.

**Supplementary Figure 6 shows the flow cytometry gating strategy for colonic lamina propria mononuclear cells, as outlined in Fig. 5 and Supplementary Figures 7 and 17**

Flow cytometry gating was used to assess CD4^+^ and CD8^+^ T cell subsets, such as CD4^+^ IL-4^+^, CD25^+^ FOXP3^+^, CD4^+^ IFN-γ^+^, and CD4^+^ IL-17A^+^, in colonic lamina propria mononuclear cells (LPMCs) from mice exposed to cigarette smoke or those with colitis induced by TNBS or DSS.

**Supplementary Figure 7 GPR15 is linked to colitis in smoke-exposed mice, with unchanged Treg cell proportions in the TNBS model and no significant changes in the proportion of Th17 cells in the DSS model**

**a** Flow cytometry gating method for identifying CD25^+^ FOXP3^+^ (Treg) cells in PBMCs (top), MLNs (middle), and LPMCs (bottom) across experimental groups in the TNBS model. The proportions of Treg cells in PBMCs **b**, MLNs **c**, and LPMCs **d** were quantified using flow cytometry. **e** Flow cytometry diagrams detailing the detection strategies for CD4^+^ IL17A^+^ (Th17) cells in PBMCs (top), MLNs (middle), and LPMCs (bottom) across different DSS model groups. Flow cytometry analysis was conducted to evaluate the levels of Th17 cells in PBMCs **f**, MLNs **g**, and **h** LPMCs. Each group had six mice. The data are presented as the means ± SEMs (n=6); **p* < 0.05, ***p* < 0.01, and ****p* < 0.001 by the two-way ANOVA with multiple comparisons. All the experiments were repeated three times, producing consistent results.

**Supplementary Figure 8 GPR15 has no effect on the secretion of IL-4 and IFN-γ in mice with TNBS- or DSS-induced colitis under cigarette smoke exposure**

**a** IL-10 and **b** TGF-β levels in colon tissue homogenates were measured using ELISA in *Gpr15*^+/+^ and *Gpr15*^-/-^ mice with TNBS-induced colitis, both with and without cigarette smoke exposure. **c** IL-17 and **d** IL-6 levels were similarly evaluated in *Gpr15*^+/+^ and *Gpr15*^-/-^ mice with DSS-induced colitis under identical conditions. **e** IL-4 and **f** IFN-γ levels in colon tissue homogenates were measured by ELISA in *Gpr15*^+/+^ and *Gpr15*^-/-^ mice under TNBS-induced colitis, with or without cigarette smoke exposure. **g** IL-4 and **h** IFN-γ levels were similarly measured in *Gpr15*^+/+^ and *Gpr15*^-/-^mice with DSS-induced colitis under the same conditions. **i** Expression levels of *IL-4* and **j** *IFN-γ* mRNA in colonic tissues of CD patients with or without cigarette smoke exposure (n=5). **k** The mRNA expression levels of *IL-4* and **l** *IFN-γ* in UC group are shown (n=5). The data are presented as the means ± SEMs. Each group contained six mice. Statistical significance was assessed using two-way ANOVA with multiple comparisons (**p* < 0.05, ***p* < 0.01, ****p* < 0.001). All the experiments were performed in three biological replicates with consistent results.

**Supplementary Figure 9 Functional impact of GPR15 on Th17 and Treg cell responses in adoptive transfer experiments**

**a** Transferred Th17 cells from cigarette smoke-treated *Gpr15*^+/+^ and *Gpr15*^-/-^ mice into *Gpr15*^-/-^ mice with TNBS-induced colitis resulted in changes in body weight, DAI scores, and colon length. **b** Transferred Treg cells from cigarette smoke-treated *Gpr15*^+/+^ and *Gpr15*^-/-^ mice into *Gpr15*^-/-^ mice with DSS-induced colitis resulted in changes in body weight, DAI scores, and colon length. **c** Representative images of colon sections from the TNBS model: left side showing H&E staining and right side displaying colonoscopy images. **d** Representative images of colon sections from the DSS model: left side showing H&E staining and right side displaying colonoscopy images. **e** TNBS model illustrating histology scores on the left, MEICS in the middle, and *Il-6* mRNA expression levels on the right. **f** DSS model illustrating histology scores on the left, MEICS in the middle, and *Il-6* mRNA expression levels on the right. Scale bar, 100 μm. Each group comprised six male mice. The data are presented as the means ± SEMs; **p* < 0.05, ***p* < 0.01, and ****p* < 0.001 by two-way ANOVA with multiple comparisons. All the experiments were conducted independently in three biological replicates, and yielded consistent results.

**Supplementary Figure 10 depicts the flow cytometry gating strategy for identifying T cell subsets from sorted naïve CD4^+^ T cells *in vitro*, detailed in Fig. 7 and Supplementary Figure 12**

Flow cytometry was utilized to analyze T cell subsets (CD25^+^ FOXP3^+^, CD4^+^ IL-17A^+^, CD25^+^ FOXP3^+^ GPR15^+^, CD4^+^ IL-17A^+^ GPR15^+^, CD4^+^ IFN-γ^+^ and CD4^+^ IL-4^+^) differentiated from sorted naïve CD4^+^ T cells isolated from the spleens of *Gpr15*^+/+^ and *Gpr15*^-/-^ mice following *in vitro* exposure to cigarette smoke extract (CSE).

**Supplementary Figure 11 CSE-mediated phosphorylation dynamics of STAT3, SMAD3, and STAT5 in *Gpr15*-dependent naïve CD4⁺ T cell differentiation**

**a** Western blot analysis demonstrating the phosphorylation status of STAT3 (p-STAT3) and total STAT3 in naïve CD4⁺ T cells from *Gpr15*^+/+^ and *Gpr15*^-/-^ mice subjected to CSE exposure (1%) for 0, 12, and 24 hours during Th17 differentiation. β-actin served as a loading control. **b** Western blot analysis illustrating the phosphorylation status of STAT5 (p-STAT5) and SMAD3 (p-SMAD3), along with total levels of STAT5 and SMAD3 in naïve CD4⁺ T cells from *Gpr15*^+/+^ and *Gpr15*^-/-^ mice exposed to CSE (1%) for 0, 12, and 24 hours during iTreg differentiation. β-actin served as a loading control. All the experiments were performed in three biological replicates, yielding consistent results (n=3).

**Supplementary Figure 12 Exposure to CSE fails to promote the differentiation of naïve CD4+ T cells into Th17 and iTreg cells *in vitro* in the absence of *Gpr15***

**a** Representative gating strategy for CD4^+^ IL-17A^+^ (Th17) cells derived from naïve CD4^+^ T cells isolated from *Gpr15*^-/-^ mouse spleens and cultured with varying concentrations of CSE. **b** Flow cytometry analysis illustrating the gating strategy for CD25^+^ FOXP3^+^ (iTreg) cells generated from naïve CD4^+^ T cells isolated from *Gpr15*^-/-^ mouse spleens. **c** Quantification of Th17 cells in stimulated naïve CD4^+^ T cells cultured with varying concentrations of CSE. **d** Comparison of iTreg cell frequencies among different groups. **e** Relative mRNA expression of *Il-17a* in CSE-treated Th17 cells. **f** Relative mRNA expression of *Foxp3* in CSE-treated iTreg cells. **g** Flow cytometry was used to evaluate IFN-γ^+^ CD4^+^ (Th1) and **h** IL-4^+^ CD4^+^ (Th2) cell populations differentiated from naïve CD4^+^ T cells isolated from *Gpr15*^+/+^ mice spleens and cultured with varying concentrations of CSE. **i** Frequencies of IFN-γ^+^ CD4^+^ T cells across groups. **j** Frequencies of IL-4^+^ CD4^+^ T cells across groups. **k** Relative *Ifn-γ* mRNA expression in CSE-treated Th1 cells. **l** Relative *Il-4* mRNA expression in CSE-treated Th2 cells. The data are presented as the means ± SEMs (n=3); **p* < 0.05, ***p* < 0.01 and **p* < 0.001 by the two-way ANOVA with multiple comparisons. All the experiments were performed in three biological replicates with reproducible results.

**Supplementary Figure 13 a, c** Colon tissue was collected post-experiment, with representative images from each group displayed, featuring a 1 cm scale bar. **b, d** Cotinine levels in murine urine. The data are presented as the means ± SEMs (n=6). Statistical significance was determined using the two-way ANOVA with multiple comparisons (**p* < 0.05, ***p* < 0.01, ****p* < 0.001). All the experiments were performed in three biological replicates with consistent results.

**Supplementary Figure 14 *GPR15* mRNA expression levels in Caco-2 and HcoEpi cell lines following exposure to various concentrations of CSE**

**a** *GPR15* mRNA expression levels in Caco-2 cell lines following exposure to various concentrations of CSE. **b** *GPR15* mRNA expression levels in HcoEpi cell lines following exposure to various concentrations of CSE. The data are presented as the means ± SEMs (n=3); no significant differences were observed, as determined by two-way ANOVA with multiple comparisons. All the experiments were conducted in three biological replicates, yielding consistent results.

**Supplementary Figure 15 Nicotine interventions in TNBS and DSS-induced mouse models of colitis**

**a** Experimental timelines for the TNBS models, illustrating treatment with vehicle or nicotine through drinking water at 200 µg/mL. **b** Experimental timelines for the DSS models, illustrating treatment with vehicle or nicotine through drinking water at 200 µg/mL. **c** Changes in body weight, DAI scores, and colon length in the TNBS model. **d** Changes in body weight, DAI scores, and colon length in the DSS model. **e** Colon images from the TNBS model: H&E staining with the histology score in the bottom-left of the left panel. **f** Colon images from the DSS model: H&E staining with the histology score in the bottom-left corner of the right panel. **g** Colonoscopy images for the TNBS group with MEICS shown in the bottom-right of the left panel. **h** Colonoscopy images for the DSS group with MEICS shown in the bottom-right of the right panel. **i** mRNA expression levels of *Il-1β*, *Il-6*, and *Tnf-α* in the TNBS group (left). **j** mRNA expression levels of *Il-1β* , *Il-6*, and *Tnf-α* in the DSS group (right). Scale bar: 100 μm. The data are presented as the means ± SEMs (n=6); **p* < 0.05, ***p* < 0.01, **p* < 0.001 (two-way ANOVA with multiple comparisons). All the experiments were carried out in three biological replicates, leading to consistent findings.

**Supplementary Figure 16 C10orf99 ligand intervention in TNBS and DSS-induced mouse models of colitis**

**a** Experimental timeline of the TNBS model outlines the administration of C10orf99 (GPR15L) at a dosage of 1 mg/kg via oral gavage (i.g.). **b** Experimental timeline of the DSS model outlines the administration of C10orf99 (GPR15L) at a dosage of 1 mg/kg via oral gavage (i.g.). **c** Percentage changes in body weight, DAI scores, and colon length in the TNBS colitis model. **d** Percentage changes in body weight, DAI scores, and colon length in the DSS colitis model. **e** Colon images from the TNBS model: H&E staining; bottom-left shows the histology score. **f** Colon images from the DSS model: H&E staining; bottom-right shows the histology score. **g** Colonoscopy images for the TNBS group are shown in the left panel, with the corresponding MEICS displayed in the bottom-right region of this panel. **h** Colonoscopy images for the DSS group are shown in the right panel, with the corresponding MEICS displayed in the bottom-right region of this panel. **i** mRNA expression levels of *Il-1β*, *Il-6*, and *Tnf-α* in the TNBS model group are shown (left). **j** mRNA expression levels of *Il-1β*, *Il-6*, and *Tnf-α* in the DSS model group are shown (right). Scale bar, 100 μm. Each group consisted of six male mice. The data are presented as the means ± SEMs; ns (not significant), **p* < 0.05, ***p* < 0.01, and ****p* < 0.001 by the two-way ANOVA with multiple comparisons. All the experiments utilized three biological replicates, yielding consistent results.

**Supplementary Figure 17 GPR15 does not influence Th1/Th2 cell differentiation in TNBS and DSS-induced colitis in mice exposed to cigarette smoke**

**a** IFN-γ expression in CD4^+^ T cells isolated from LPMCs was evaluated by flow cytometry in the TNBS-induced colitis model. **b** Comparative analysis of IFN-γ^+^ CD4^+^ T (Th1) cell populations across all groups. **c** Gating strategy for IL-4 expression in CD4^+^ T cells from LPMCs in TNBS-induced colitis. **d** Frequency of IL-4^+^ CD4^+^ T (Th2) cells across all groups. **e** Gating strategy for identifying IFN-γ⁺ CD4⁺ (Th1) T cell populations in LPMCs in the DSS-induced colitis model. **f** IFN-γ^+^ CD4^+^ T cell populations across all groups. **g** Gating strategy for IL-4^+^ CD4^+^ T (Th2) cells in LPMCs in the DSS-induced colitis model. **h** Percentages of IL-4^+^ CD4^+^ T cells in each group. Each group consisted of six mice. The data are presented as the means ± SEMs; statistical significance is indicated as **p* < 0.05, ***p* < 0.01, and **p* < 0.001 using the two-way ANOVA with multiple comparisons. All the experiments were performed three times, yielding consistent results.

**Supplementary Figure 18 Generation and genotyping *Gpr15*^-/-^ mice**

**a** Schematic diagram showing the method for generating *Gpr15* knockout mice. A *Gpr15* targeting vector was constructed with two LoxP sites flanking the 5' ATG start codon and 3' TAA stop codon in the same direction. The application of Cre recombinase, which interacts with the LoxP sites, resulted in a *Gpr15* loss-of-function allele. **b** DNA genotyping confirmed the *Gpr15* knockout mice. PCR analysis with oligonucleotide primers forward 1 and reverse 1 identified bands at 625 bp and 1579 bp, indicating the wild-type allele. Conversely, the same primer set yielded a 601 bp band, signifying the deletion of the *Gpr15* allele. Furthermore, using a different primer set, forward 2 and reverse 2, resulted in a 625 bp band alongside the product from primer set 1, confirming the heterozygosity. Lanes 1-2, positioned directly after the marker, without adding DNA template served as a negative control. Lanes 3-4 display DNA extracted from *Gpr15*^+/-^ mice, lanes 5-6 from *Gpr15*^+/+^ mice, and lanes 7-8 from *Gpr15*^-/-^ mice. **c** Colonic mRNA expression in *Gpr15*^+/+^, *Gpr15*^+/-^, and *Gpr15*^-/-^ mice. For sequences of the oligonucleotide primers, please refer to Table 4.

**Supplementary Figure 19 Generation of** **C57BL/6JGpt-Tg(human *GPR15*) transgenic mice**

**a** Strategy diagram for generating a transgenic mouse model. The human BAC clone *RP11-349K13,* containing the human *GPR15* locus, was selected for generating a transgenic mouse model. The BAC vector was modified *in vitro* to retain the 10 kb upstream promoter/enhancer regions and 2.0 kb downstream sequences flanking the *GPR15* gene. This ensures the preservation of endogenous regulatory elements and maintains native expression patterns. The construct was microinjected into pronuclei of C57BL/6JGpt zygotes and integration as well as desired structure confirmed in F0 mice by PCR. **b** The PCR analysis shows bands confirming the correct insertion of the BAC targeting construct. Lanes 1 and 2 are wild-type controls, lanes 3 and 4 show positive integration and lanes 5 and 6 are negative controls. Primer sequences are listed in Table 5. **c** Quantitative PCR shows the relative expression of the *GPR15* transgene in mouse colon tissue. Small amounts of expression in the wild-type control could be due to background detection of the endogenous mouse *Gpr15* mRNA by partially matching primers.

| **Supplementary Table 1 Associations between smoking status and inflammatory bowel disease** | | | | | | |
| --- | --- | --- | --- | --- | --- | --- |
| **CD(n=2120)** | | | | | | All Age |
| **State** | **HR** | **lower** | **upper** | ***p*** | **n(CD+&State+)** |  |
| Never Smoking | Ref | | | | |  |
| Ever Smoking | 1.2719 | 1.1636 | 1.3903 | 1.19E-07 | 1381 |  |
| Previous Smoking | 1.2655 | 1.1554 | 1.3869 | 3.98E-07 | 840 |  |
| Current Smoking | 1.3707 | 1.2114 | 1.5509 | 5.62E-07 | 306 |  |
| **UC(n=4161)** | | | | | |  |
| **State** | **HR** | **lower** | **upper** | ***p*** | **n(UC+&State+)** |  |
| Never Smoking | Ref | | | | |  |
| Ever Smoking | 1.2084 | 1.1324 | 1.2894 | 1.11E-08 | 2708 |  |
| Previous Smoking | 1.4795 | 1.3897 | 1.5752 | 2.00E-16 | 1867 |  |
| Current Smoking | 0.7569 | 0.6788 | 0.844 | 5.36E-07 | 348 |  |

CD: Crohn’s Disease; UC: Ulcerative Colitis

**Supplementary Table 2. Multivariable and Interaction Analyses of Smoking Status and GPR15-Proximal SNPs in Relation to Ulcerative Colitis and Crohn’s Disease Risk in the UK Biobank**

| **Disease** | **Smoking Status** | **Statistical Model** | **HR (95% CI)** | ***p*-value** | **Selected SNP(s)** |
| --- | --- | --- | --- | --- | --- |
| UC | Ever smoking | Adjusted model | 1.22 (1.14-1.30) | 7.86E-09 | - |
|  |  | Adjusted + SNPs | 1.23 (1.15-1.32) | 6.91E-09 | chr3-97703142-C-T, chr3-97790053-G-*, chr3-99319111-A-G, chr3-99363401-A-G |
| UC | Previous smoking | Adjusted model | 1.49 (1.40-1.59) | 1.91E-34 | - |
|  |  | Adjusted + SNPs | 1.56 (1.45-1.68) | 8.15E-34 | chr3-97703142-C-T, chr3-97790053-G-*, chr3-98886876-T-C, chr3-99363401-A-G, chr3-99451539-C-T, chr3-99451539-C-T |
| UC | Current smoking | Adjusted model | 0.75 (0.67-0.84) | 1.09E-06 | - |
|  |  | Adjusted + SNPs | 0.70 (0.62-0.80) | 1.83E-07 | chr3-97630944-C-T, chr3-97703142-C-T, chr3-98033434-A-G, chr3-98327216-T-C, chr3-98407130-C-T, chr3-98435846-C-T, chr3-98652371-G-A, chr3-98750373-A-G, chr3-98886876-T-C, chr3-98999931-T-G, chr3-99319111-A-G, chr3-99363401-A-G, chr3-99451539-C-T, chr3-99451539-C-T |
| CD | Ever smoking | Adjusted model | 1.28 (1.16-1.40) | 3.05E-07 | - |
|  |  | Adjusted + SNPs | 1.27 (1.16-1.40) | 4.30E-07 | chr3-98535927-T-* |
| CD | Previous smoking | Adjusted model | 1.29 (1.18-1.41) | 4.97E-08 | - |
|  |  | Adjusted + SNPs | 1.29 (1.18-1.41) | 5.20E-08 | chr3-98535927-T-* |
| CD | Current smoking | Adjusted model | 1.34 (1.18-1.52) | 4.81E-06 | - |
|  |  | Adjusted + SNPs | 1.35 (1.19-1.53) | 2.85E-06 | chr3-98535927-T-* |

Hazard ratios (HRs) and 95% confidence intervals (CIs) are shown for each disease, smoking status, and model. “Adjusted + SNPs” models additionally include forward-selected SNPs near GPR15 (chr:position-reference allele-variant allele) based on statistical significance and independence. *p*-values are given in scientific notation (e.g., 8.15E-34 = 8.15 × 10⁻³⁴). An asterisk (*) indicates a non-specific or alternative allele at the SNP site.

| **Supplementary Table 3 Sample characteristics for IBD** | |  |
| --- | --- | --- |
| IBD subtypes | No. |  |
| CD | 10 |  |
| Colonic | 6 |  |
| Small bowel | 4 |  |
| UC | 10 |  |
| Sex |  |  |
| Male | 11 |  |
| Female | 9 |  |
| Smoking |  |  |
| Current | 10 |  |
| Never | 10 |  |
| Quit | 0 |  |
| CD: Crohn’s Disease; UC: Ulcerative Colitis |  |  |

| **Supplementary Table 4 Primer sequences used for PCR in *Gpr15*^-/-^ mice** | |
| --- | --- |
| genes |  |
| F1 | 5’-CCACAACCACAGCCAATACCAATC-3’ |
| R1 | 5’-TGAGTTCAAAGACAGTGGAGTTTG-3’ |
| F2 | 5’-GATCGTTATTATTGCGGTGGCGG-3’ |
| R1 | 5’-TGAGTTCAAAGACAGTGGAGTTTG-3’ |

F: Forward; R: Reverse

| **Supplementary Table 5 Primer sequences used for PCR in C57BL/6JGpt-Tg (human *GPR15*) mice** | |
| --- | --- |
| genes |  |
| F1 | 5’-CAGATAAGGTCATGGACAGCATTGG-3’ |
| R1 | 5’-AAGGCATTAGGGGACAGAGTGG-3’ |
| F2 | 5’-AAGGAAGCTGTGTGCCCATTACC-3’ |
| R2 | 5’-AAGGGCACAAGCAGTGGACAAT-3’ |

F1: GPT00CR01003-01-h*GPR15*-tF1; R1: GPT00CR01003-01- h*GPR15*-tR1

F2: GPT00CR01003-01-h*GPR15*-tF2; R2: GPT00CR01003-01- h*GPR15*-tR2

| **Supplementary Table 6 RT-qPCR primers for mRNA expression used in this study** | | |
| --- | --- | --- |
| **Gene symbol** | **Primer sequence (5’-3’)** | **PCR product size (bp)** |
| Mouse *β-actin*-F | TGCTTCTAGGCGGACTGTTAC | 233 |
| Mouse *β-actin*-R | AACCAACTGCTGTCGCCTT |  |
| Mouse *Tnf-α*-F | AGGCACTCCCCCAAAAGATG | 213 |
| Mouse *Tnf-α*-R | CCACTTGGTGGTTTGTGAGTG |  |
| Mouse *Il-1β*-F | TGCCACCTTTTGACAGTGATG | 220 |
| Mouse *Il-1β*-R | AAGGTCCACGGGAAAGACAC |  |
| Mouse *Il-6*-F | CAACGATGATGCACTTGCAGA | 251 |
| Mouse *Il-6*-R | TGTGACTCCAGCTTATCTCTTGG |  |
| Human *IL-17A*-F | CCCCTAGACTCAGGCTTCCT | 153 |
| Human *IL-17A*-R | AGTTCGTTCTGCCCCATCAG |  |
| Human *IL-10*-F | CACATCAGGGGCTTGCTCTT | 151 |
| Human *IL-10*-R | GGTGCAGCTGTTCTCAGACT |  |
| Human *TGF-β*-F | ACCTGCCACAGATCCCCTAT | 712 |
| Human *TGF-β*-R | CCGGTAGTGAACCCGTTGAT |  |
| Human *IL-6*-F | CCTTCGGTCCAGTTGCCTT | 570 |
| Human *IL-6*-R | AGCTGCGCAGAATGAGATGA |  |
| Human *IL-4*-F | CTTTGCTGCCTCCAAGAACAC | 97 |
| Human *IL-4*-R | GCGAGTGTCCTTCTCATGGT |  |
| Human *β-actin*-F | CACGATGTACCCTGGGATCG | 118 |
| Human *β-actin-*R | GCCGATCCACACCGAGTATT |  |
| Human *IFN-γ*-F | CAGCTCTGCATCGTTTTGGG | 418 |
| Human *IFN-γ*-R | CTGTTTTAGCTGCTGGCGAC |  |
| Human *GPR15*-F | TTGCATTTCAAACCCGGCAG | 133 |
| Human *GPR15*-R | CCGTCCTCCACAGTCCTAGA |  |

F: Forward; R: Reverse
